# Clonal dynamics shaped by diverse drug-tolerant persister states in melanoma resistance

**DOI:** 10.1186/s12943-026-02622-9

**Published:** 2026-03-03

**Authors:** Haiyin Li, Yeqing Chen, Jessica Kaster, Maggie Dunne, Min Xiao, Ling Li, Monzy Thomas, Nazifa Promi, Dylan Fingerman, Gregory Schuyler Brown, Qiuxian Zheng, Xingyue Zhu, McKenna Reale, Andrew Patterson, Le Gao, Xuxiang Zhang, Siqi Jiang, Tianxing Hu, Hanzhang Fang, Jianlan Ren, Cong Qi, Luyang Wang, Haiwei Mou, Gatha Thacker, Eric Ramirez Salazar, Jessie Villanueva, Arjun Raj, Dave SB Hoon, Tian Bin, Jozef Madzo, Zhi Wei, Noam Auslander, Meenhard Herlyn

**Affiliations:** 1 The Wistar Institute, Philadelphia, PA USA; 2 Department of Computer Science, New Jersey Institute of Technology, Newark, NJ USA; 3 Department of Bioengineering, School of Engineering and Applied Sciences, University of Pennsylvania, Philadelphia, PA USA; 4 Department of Genetics, Perelman School of Medicine, University of Pennsylvania, Philadelphia, PA USA; 5 St John’s Cancer Institute, Santa Monica, CA USA

**Keywords:** Lineage tracing, Drug-tolerant persister cells, Melanoma therapy resistance, Cancer plasticity, Tumor heterogeneity, Clonal dynamics, Single-cell transcriptomics, Spatial transcriptomics

## Abstract

**Background:**

Most advanced melanomas initially respond to targeted therapy but eventually relapse. Increasing evidence suggests that drug-tolerant persister cells can adopt a reversible drug-refractory state and represent a key driver of therapeutic resistance.

**Methods:**

We developed MeRLin, a lineage tracing platform that integrates cellular barcoding, single-cell transcriptomic profiling, RNA fluorescence in situ hybridization, and computational analyses to track clonal and transcriptional dynamics in a patient-derived melanoma model during prolonged targeted therapy. Longitudinal analyses enabled the characterization of clonal fates, transcriptional states, and spatial organization of persister populations.

**Results:**

Clonal dynamics showed that persister subpopulations initially responded to therapy, persisted through minimal residual disease, and expanded during tumor recurrence. Four persister-associated transcriptional states characterized by stress-like, lipid metabolism, PI3K signaling, and extracellular matrix remodeling programs were associated with persister populations arising from minor pre-treatment subpopulations under sustained drug pressure. Spatial transcriptomic analyses revealed structured spatial organization of these programs and suggested coordinated autocrine and paracrine interactions among persister states. Targeted barcode RNA fluorescence in situ hybridization enabled spatial mapping of clonal identity and gene expression, revealing in situ co-localization of a dominant resistant clone marked by *SLC2A1* expression.

**Conclusions:**

Together, MeRLin provides a robust framework for dissecting cancer heterogeneity and characterizing persister subpopulations. Our findings demonstrate that melanoma recurrence is associated with diverse, spatially organized persister states linked to adaptive transcriptional programs.

**Supplementary Information:**

The online version contains supplementary material available at 10.1186/s12943-026-02622-9.

## Introduction

Despite initially exhibiting strong responses to targeted therapy, the majority of tumors eventually relapse, often without gaining new resistance mutations [1, 2]. The presence of drug-tolerant persister (DTP) cells, a subpopulation of cancer cells capable of entering a reversible drug-refractory state, contribute to minimal residual disease (MRD) [3]. DTP-mediated resistance stems from intratumoral phenotypic heterogeneity driven by diverse gene expression programs and adaptive survival mechanisms [3]. During treatment, cancer cells may transiently activate survival pathways and resistance genes, entering a DTP state [3]. Prolonged drug exposure enables DTP cells to develop diverse, heritable resistance, allowing them to tolerate cytotoxic stress, establish MRD, and seed subsequent outgrowth of resistant clones^4^. Understanding the mechanisms of DTP formation, plasticity, and heterogeneity in cancer is crucial for the discovery of resistance markers and therapeutic targets [3]. The detection and targeting of DTP cells may offer novel strategies to overcome resistance and improve long-term treatment efficacy [1, 5].

Lineage tracing technologies provide critical insight into tumor heterogeneity and clonal evolution [6]. The Confetti system uses Cre/lox-mediated stochastic recombination of fluorescent reporters to visualize clonal expansion and dissemination in vivo [7], while the lentiviral-based LeGO system enables scalable color barcoding and quantitative tracking of clonal diversity in xenograft models [8]. Static barcoding strategies such as LARRY and CellTag allow clonal labeling that can be read out by single-cell RNA sequencing (scRNA-seq) [9, 10], and the Watermelon system further links clonal identity with cell state by combining lentiviral barcodes, fluorescent proliferation reporters, and transcriptomic profiling [11]. In parallel, methods such as FateMap [4], derived from Rewind [12], and imaging-compatible lineage recorders using sequential rounds of fluorescence in situ hybridization (FISH)-based hybridization, including intMEMOIR [13], baseMEMOIR [14], and PEtracer [15], have expanded the ability to reconstruct clonal relationships with spatial context alongside gene expression analysis. Here, we developed MeRLin (Melanoma Resistance Lineage tracing), a single-vector, high-complexity static barcoding platform designed for cancer xenograft models. MeRLin integrates clonal tracking with single-cell transcriptomic profiling and enables targeted spatial validation of selected clones using standard RNA-FISH. It also allows in vivo imaging through bioluminescence and far-red fluorescence. This unified design supports the integrated analysis of clonal dynamics, transcriptional plasticity, and spatial organization during melanoma progression and therapeutic response.

Two-dimensional (2D) melanoma cell culture remains a foundational model for high-throughput drug screening and molecular characterization but lacks the heterogeneity and physiological complexity of in vivo tumors [16]. Patient-derived xenograft (PDX) models, in which tumor tissues from patients are implanted into immunocompromised or humanized mice, more accurately recapitulate spatial structure of cancer and intratumor heterogeneity [17]. PDX models retain the genomic features of patients across different stages, subtypes, and diversified treatment backgrounds, making them indispensable for studying therapeutic response and resistance mechanisms [17].

Using MeRLin in a melanoma PDX model that recapitulates the clinical development of BRAF/MEK inhibitor (BRAFi/MEKi) resistance, we traced the clonal origins and transcriptional states of thousands of individual melanoma cells as resistance developed in vivo. Prolonged targeted therapy was associated with the presence of molecularly and functionally diverse drug-tolerant persister states arising from minor pre-treatment subpopulations, characterized by four adaptive transcriptional programs. We identified genes enriched within persister subpopulations and spatially mapped a dominant resistant clone marked by *SLC2A1* expression in recurrent tumors, providing proof-of-principle validation of targeted spatial clonal analysis. Together, these findings delineate transcriptional programs and candidate pathways associated with drug-tolerant persister formation, plasticity, and heterogeneity in melanoma resistance.

## Methods

### Cloning of the MeRLin library

Starting with the pCDH-EF1a-eFFly-mCherry plasmid [18] (Addgene #104833, a gift from Irmela Jeremias), we derived a lentiviral vector backbone pCDH-EF1a-eFFly-mNeptune2.5 by replacing mCherry with mNeptune2.5 from pcDNA3-mNeptune2.5 [19] (Addgene #51310, a gift from Michael Lin), using NsiI and SalI restriction sites (NEB) (Supplementary Table 1). To optimize the simultaneous sequencing of barcodes by scRNA-seq, we used the Q5 Site-Directed Mutagenesis Kit (NEB E0552) to mutate the original KpnI site in the 3’ LTR (C-to-T at position 3931) and introduce a new KpnI site (6-nt insertion at position 3961). For barcode insertion, we ordered PAGE-purified Ultramer oligonucleotides (IDT) of barcode inserts containing semi-random repeat patterns and homologous flanks (Table S1). Barcode insert 1 contained 60-nt repeats of ‘WWSS’ (W = A/T, S = G/C), 60-nt repeats of ‘NSW’ (N = any base), and a 25-nt gap sequence. Barcode insert 2 contained the same 25-nt gap, followed by 60-nt repeats of ‘WS’ and 60-nt repeats of ‘SNW’. Barcode insert 1 had a 28-nt left flank, while barcode insert 2 had a 33-nt right flank homologous to the vector insertion site for Gibson Assembly. We digested the vector for 5 h with KpnI (NEB), gel-purified the linearized vector, and performed Gibson Assembly using 30 fmol of vector, 160 fmol of barcode oligonucleotides (100 nM in nuclease-free water), 15 µl of NEBuilder HiFi DNA Assembly Master Mix (NEB E2621), and nuclease-free water to a final volume of 30 µl, incubating at 50°C for 1 h. The assembled plasmid was column-purified (Monarch PCR & DNA Cleanup Kit, NEB T1030) and then 25 ng of the column-purified plasmid was electroporated into 25 µl of Endura Electrocompetent Cells (Lucigen 60242-1) using a Gene Pulser II (Bio-Rad) under the following conditions: 10 µF capacitance, 600 Ohms resistance, 1.8 kV voltage, and a time constant of ~ 5–6 ms. We performed nine parallel electroporations. Immediately after electroporation, we added 975 µl of pre-warmed (37°C) recovery media to each electroporation cuvette and then transferred the liquid to 1.5-ml microcentrifuge tubes and placed these tubes on a shaker at 220 rpm and 37°C for 1 h. After recovery, we took 10 µl of the culture for plating serial dilutions. The rest of the cultures were pooled (4–5 electroporation per batch) into 150 ml LB broth containing 100 µg/ml ampicillin, and incubated at 30°C, 220 rpm for 12–14 h before plasmid isolation (EndoFree Plasmid Maxi Kit, Qiagen). Some pellets were stored at − 20°C before isolation. Transformation efficiency was estimated by colony counting from the plated serial dilutions, and barcode insertion was verified via polymerase chain reaction (PCR) on randomly picked 20–30 colonies using DreamTaq Green PCR Master Mix (Thermo Fisher Scientific, K1081). Plasmid sub-libraries were pooled before lentivirus packaging. The final barcode library diversity of MeRLin was estimated at 2.89 million (Table S2).

### Cell culture

Melanoma WM4237-1 cell line was established in our lab, and cultured in TU2% media consisting of 80% MCDB 153, 10% Leibovitz’s L-15, 2% FBS, 2.4 mM CaCl2, and passaged using 0.05% trypsin-EDTA.

For lentivirus packaging, we cultured Lenti-X 293T cells (Clontech) in DMEM containing 10% FBS, and similarly passaged for lentivirus production.

### Lentivirus packaging and transduction

Before plasmid transfection, Lenti-X 293T cells were grown to ~ 80% confluency in 10 cm tissue culture dishes in DMEM containing 10% FBS. For each dish, the medium was replaced with 10 mL of DMEM (10% FBS) containing 25 µM chloroquine diphosphate, followed by a 5-hour incubation. For transfection, 81.6 µl of Transporter 5 (Polysciences) was added to 420 µl of Opti-MEM (Thermo Fisher Scientific, 31985062). In parallel, 8.6 µg of psPAX2, 2.6 µg of pMD2.G, and 9.2 µg of the barcode plasmid library were mixed in 500 µl of Opti-MEM. Diluted Transporter 5 was added dropwise to the diluted DNA tube, inverted and incubated for 20 min before being added dropwise to the culture dish. Approximately 18 h post-transfection, the medium was aspirated, cells were washed once with 1× DPBS, and fresh DMEM (5% FBS) was added. Virus-containing media were collected at 48 and 72 h post-transfection and stored at 4 °C. After the final collection, pooled virus-laden media were centrifuged at 500 × g for 5 min and filtered through a 0.45 μm PES filter. To concentrate the lentivirus, four volumes of virus-containing media were mixed with one volume of cold Lenti Concentrator (Origene, TR30025) and incubated overnight at 4 °C with constant rocking. The concentrated lentiviral particles were pelleted by centrifugation at 3,000 × g for 35 min at 4 °C and resuspended in cold, sterile 1× DPBS at 1/20th of the original media volume by gentle pipetting. The purified lentivirus was aliquoted and stored at -80 °C.

To transduce melanoma cells, freshly thawed lentiviral library and polybrene (final concentration 8 µg/mL) were added to dissociated cells, which were then plated in six-well plates (225,000 cells in 1 mL of media per well) and centrifuged at 600 × g for 30 min at 32 °C. After overnight incubation at 37 °C, the media was removed, cells were washed once with 1× DPBS, and 2 mL of TU2% was added to each well. Cells were then passaged into 10-cm dishes in the above-mentioned media. Dissociated cells were sorted by flow cytometry for mNeptune2.5-positive cells to achieve a multiplicity of infection (MOI) below 0.4. Barcoded cells were expanded and divided into replicates, with one replicate saved as the original control *In vitro*. 

### Drug treatment

Stock solutions of 10 mM dabrafenib (NSC 764134, NCI) and 100 µM trametinib (NSC 758246, NCI) were freshly prepared in DMSO. For drug treatment, stock solutions were diluted in culture medium to final concentrations of 0.3 µM for dabrafenib and 30 nM for trametinib. WM4237-1 cells were treated for one week, with either DMSO (vehicle control) or drug-containing medium refreshed every two days. Three replicates were pooled for both control and treatment conditions. Cells were trypsinized, washed, pelleted, and resuspended in 100 µl dead cell removal antibody per 10 million cells (Miltenyi Biotec, Dead Cell Removal Kit). Following a 15-minute incubation at room temperature, LS columns were primed with 3 ml 1× dead cell removal buffer, and cells were washed with 2 ml buffer per 10 million cells, followed by four additional washes with 3 ml buffer. A final centrifugation at 250 × g for 5 min was performed, the supernatant was completely removed, and the pellet was resuspended in 80 µl PBS before transferring to an Eppendorf tube on ice. A final cell count was performed before proceeding to bulk RNA-seq and scRNA-seq.

### Animal studies

All animal procedures were approved by the Institutional Animal Care and Use Committee (IACUC, #201546). For injections of barcoded WM4237-1 cells, 6-8-week-old male immunodeficient NSG mice were shaved on their lower back and injected subcutaneously into the right flanks (5 × 10⁵ in 100 µL RPMI: Matrigel (Corning), 1:1). The mice were received from the in-house breeding facility and housed under pathogen-free conditions. Tumor size monitoring was performed using caliper measurement and calculated using the following formula volume = (W^2^ × L)/2, where W and L refer to the short and long tumor diameter, respectively. After tumors reached a volume of ~ 500 mm^3, BRAFi/MEKi treatment was initiated with a rodent diet containing 150 mg/kg dabrafenib and 1.5 mg/kg trametinib (Bio-Serv, S7581). On Day 0, Day 21, Day 57 and day 91 (endpoint), mice were euthanized by CO2 inhalation and tumors were dissected and cut into chunks for bulk RNA-seq and formalin-fixed paraffin-embedded (FFPE) samples.

### Single-cell sample preparation

For single-cell sample preparation, tumor chunks from three replicates were pooled and minced, and 0.2–1 g of tissue was added to 5 ml dissociation solution consisting 4.7 ml RPMI with 200 µl enzyme H, 100 µl enzyme R, and 25 µl enzyme A (Miltenyi Biotec, Tumor Dissociation Kit) on ice. Samples sealed in C-tubes were proceeded onto the gentleMACS Octo Dissociator (Miltenyi Biotec) with a heating apparatus for 1 h. Following dissociation, 8 ml of serum-free media was added, and single-cell suspensions were filtered through a 70 μm nylon mesh and centrifuged at 250 × g for 7 min. The pellet was resuspended in 1 ml ACK Lysing Buffer (Quality Biological, 118-156-101), incubated for 5 min, and then diluted with 9 ml serum-free media before a second centrifugation at 250 × g for 5 min. The pellet was resuspended in 80 µl BSA buffer (0.25 g BSA in 50 ml PBS) per 2 million cells before adding 20 µl mouse antibody cocktail per 2 million cells (Miltenyi Biotec, Mouse Cell Depletion Kit). After 15 min of incubation at 4 °C, LS columns were primed with 3 ml BSA buffer, and the labeled cells were passed through, collecting the flow-through containing enriched human tumor cells. Cells were washed three times with 1 ml BSA buffer, centrifuged at 250 × g for 5 min. Dead cells were removed using the Dead Cell Removal Kit (Miltenyi Biotec) as described above. Purified human melanoma cells were resuspended in 80 µl PBS before transferring to an Eppendorf tube on ice. A cell count was conducted before proceeding to scRNA-seq.

### Bulk RNA-seq

Libraries for whole transcriptome RNA sequencing were prepared using the Stranded Total RNAseq with Ribo-zero Plus kit (Illumina, San Diego, CA) as per manufacturer’s instructions starting with an input of 700 ng of total RNA and 10 cycles of final PCR amplification. Library size was assessed using the 4200 Tapestation and the High-Sensitivity DNA assay (Agilent, Santa Clara, CA). Concentration was determined using the Qubit Fluorometer 2.0 (Thermofisher, Waltham, MA). Next Generation Sequencing with a paired-end 2 × 150 bp run length was done on the NovaSeq X platform (Illumina, San Diego, CA). A minimum of 30 M reads per sample was acquired for each sample.

### Barcoded RNA-FISH

To validate the co-localization of *SLC2A1* with the second most dominant clone (barcode suffix “GTTGAACGACCACAA”), we used paired bulk RNA-seq and scRNA-seq to recover the full-length 265 bp barcode. A custom 5ZZ antisense probe set Syn-T2v3 (ACD 1588531-C1) was designed by the ACD Probe Design Team and synthesized to target this barcode mRNA transcript for spatial mapping. Using the RNAscope™ Multiplex Fluorescent Reagent Kit v2 with TSA Vivid Dyes (ACD 323270), we visualized this persister subpopulation of barcode “GTTGAACGACCACAA” and quantified the expression of the stress-like gene *SLC2A1* (RNAscope^®^ Probe Hs-*SLC2A1*, ACD 423141-C2) in tissue sections from WM4237-1 endpoint tumor. A 5ZZ sense probe targeting the antisense sequence of barcode “GTTGAACGACCACAA” (Syn-T2v3-sense, ACD 1587501-C1) was used as the negative control as non-specific staining.

These probes were used with TSA Vivid Fluorophore 650 (ACD 323273) and TSA Vivid Fluorophore 570 (ACD 323272), respectively. The slides were then imaged using a Leica TCS SP8 X WLL scanning confocal microscope by a 63x objective lens, a zoom of 2x, and laser settings at 405 nm for DAPI staining, and 550 nm and 649 nm for the two probes. Image analysis was performed by masking, patching, and counting patches with red mask, green mask and co-localized to determine an enrichment p-value. We segmented the RNA-FISH image to 4 × 4, 6 × 6, 8 × 8, and 10 × 10 (non-overlapping) patches, to quantify co-localization of *SLC2A1* with barcode “GTTGAACGACCACAA” or non-specific control.

### Single-cell RNA-seq library preparation and read alignment

Single cell droplets were generated using the Chromium Next GEM single cell 3’ kit v3.1 (10x Genomics). cDNA synthesis and amplification, library preparation, and indexing were performed using the 10x Genomics Library Preparation kit (10x Genomics), according to the manufacturer’s instructions. The overall library size was determined using the Agilent Bioanalyzer 2100 and the high-sensitivity DNA assay, and libraries were quantitated using KAPA real-time PCR. Libraries were pooled and sequenced on the NovaSeq 6000 (Illumina, San Diego, CA, USA) using an S2 300 cycle kit (Illumina), paired end run with the following run parameters: 26 bp × 8 bp (dual index) × 280 bp.

Reads alignment was performed using Cell Ranger v8.0.0 (10x Genomics). For in vivo PDX samples, raw sequencing reads were first aligned to a combined human-mouse reference genome (refdata-gex-GRCh38_and_GRCm39-2024-A) to identify and exclude mouse-derived reads. Cells with more than 20% of the reads mapped to the mouse genome were removed. For barcoded samples, the reads were aligned to a custom reference genome that included the barcode vector sequence integrated into the human genome (GRCh38-2024-A). All the other samples were aligned directly to the human genome (GRCh38-2024-A).

### Clonal barcode extraction

Clonal barcodes and their corresponding 10x cell barcodes (CB tags) were retrieved from the BAM files generated using Cell Ranger. Reads containing barcode sequences were aligned to a pseudo-chromosome that represented the barcode vector. We have developed a clonal barcode extraction tool to automate this process. Briefly, the extractor first scans for the known 3’ sequence of the vector backbone (CAGATCTTAGCCACTTTTTAAAAGAAAAGGGGG; Table S1) to locate the start of the semi-random clonal barcode sequence in each read. The extracted clonal barcode sequences were subsequently processed through a series of quality control and correction steps, including removing empty vectors, short barcodes, barcodes with incorrect patterns, and cells with multiple barcodes. For each high-confidence barcode, the first 15 bases and their corresponding CB tags were compiled into a structured data frame (details are available on GitHub).

### Computational analysis of scRNA-seq data

All scRNA-seq expression matrices were processed using the Seurat v5 [20]. The barcode information was incorporated into the cell metadata of each Seurat object. Cells were filtered out if they had fewer than 2,000 detected genes, more than 50,000 unique molecular identifiers (UMIs), or over 20% of the reads mapped to mitochondrial genes. Doublets were identified and removed using DoubletFinder v2.0.4 [21]. Cell cycle phase scores were calculated based on the canonical S and G2/M genes. After quality control, the samples were integrated and normalized using SCTransform [22]. Principal component analysis (PCA) was performed, and the top 10 components were retained based on the elbow point of the standard deviation plot for downstream analysis.

To better resolve the clonal architecture within each scRNA-seq sample, we used ClonoCluster v0.0.1 [23], an algorithm that integrates both transcriptomic profiles and clonal barcode identities into hybrid clusters. This approach replaced standard Louvain clustering and uniform manifold approximation and projection (UMAP) in the Seurat workflow, which did not produce optimal cluster separation due to the absence of clonal barcode information in its analysis. To fine-tune the impact of clonal barcode identity on the UMAP layout, we tested various Warp Factor (WF) values and determined that WF = 6 provided the best balance between transcriptomic similarity and clonal architecture.

### Clonal barcode classification and Shannon diversity analysis

To assess the clonal dynamics of barcoded cells over time, we calculated the Shannon diversity index [24] for each sample based on the barcode abundance distributions. To classify individual barcode behaviors, we modeled their longitudinal abundance trajectories across all time points using nonlinear least squares fitting. Two competing models were tested for each barcode: an exponential decay model representing sensitive clones, and an exponential growth model representing persister clones. Barcodes best fit by the decay model (residual sum of squares, RSS < 0.1) were classified as Sensitive barcodes, whereas those better fit by the growth model (RSS < 0.1) were labeled as Persister barcodes. Barcodes that did not fit either model or remained at consistently low abundance were classified as low-abundance barcodes.

### Pseudotime trajectory inference

Pseudotime trajectory was performed using Monocle3 v.1.4.26 [25] on scRNA-seq dataset of barcoded WM4237-1 endpoint tumors. Cells were clustered using UMAP embeddings derived from the ClonoCluster pipeline with default Monocle3 clustering resolution allowing the algorithm to automatically determine optimal community structure. Trajectories were inferred using Monocle3 principal graph learning framework. The root node was manually defined at the centroid of the sensitive clonal population to represent the transcriptionally sensitive state. Pseudotime values were subsequently calculated for all cells, and pseudotime-ordered trajectories were visualized to assess transcriptional state transitions during treatment.

### Functional signature analysis by barcode group

To characterize the transcriptional programs and functional signatures associated with distinct clonal populations, we redefined the ClonoCluster-derived clusters into five barcode groups. Each group comprised a unique and mutually exclusive set of barcodes.

For each barcode group, we performed differential gene expression analysis to identify upregulated genes (log2 fold-change ≥ 1, detected in ≥ 50% of cells, and adjusted p-value < 0.05). These genes were subsequently submitted to the Enrichr platform for functional enrichment analysis [26]. Hallmark, KEGG, and Reactome databases were queried to determine biological processes associated with each group’s transcriptional program.

After defining the representative functional signatures for each group, we selected key genes from the enriched pathways and calculated signature activity scores using AUCell [27] to validate the activity of these group-specific programs at single-cell level.

### CIBERSORTx deconvolution analysis

Cell-type deconvolution of bulk RNA-seq data was performed using CIBERSORTx (https://cibersortx.stanford.edu), following the standard workflow. Single-cell RNA-seq data from WM4237 endpoint tumors were used to construct a custom reference signature matrix. Cells were grouped by barcode-defined clonal populations and used to derive representing transcriptional signatures for each subpopulation. This reference expression matrix was applied to bulk RNA-seq datasets for deconvolution to infer the relative proportions of each subpopulation across samples. CIBERSORTx was run with 1,000 permutations to enable statistical significance estimation of inferred cell fractions, and only results with significant p-values were retained in downstream analyses.

### SCENIC analysis

To infer the transcription factor activity and gene regulatory networks for each barcode group in the scRNA-seq data, we performed single-cell regulatory network inference and clustering (SCENIC) analysis using pySCENIC v0.12.1 [27]. Gene co-expression modules were first inferred using the GRNBoost2 algorithm [28]. These modules were then refined by filtering target genes based on motif enrichment within ± 10 kb of transcription start sites using the hg38 motif collection as a reference. Next, regulon activity was quantified across all cells using AUCell for each regulon. The resulting activity scores were integrated into Seurat objects to identify the top differentially active regulons across barcode types (persister, sensitive, and low-abundance) and barcode groups defined from ClonoCluster.

### Alternative polyadenylation analysis

To investigate the post-transcriptional regulation associated with treatment response, we performed alternative polyadenylation analysis (APA) using MAAPER [29] by comparing BAM files from drug treated samples to non-treated samples (day 21, day 57, endpoint vs. day 0; BRAFi/MEKi vs. CTRL). The required polyadenylation site (PAS) annotation file for hg38 was obtained from https://github.com/Vivianstats/data-pkg/tree/main/MAAPER/PolyA_DB. Genes with an adjusted p-value < 0.05 and log fold change > log (1.2) or < -log (1.2) were classified as lengthened or shortened, respectively.

### InferCNV analysis

To infer chromosomal copy number variations (CNVs) across barcode groups, we performed inferCNV v1.20.0 [30] on scRNA-seq data. The subcluster mode was used with both denoising and hidden Markov model (HMM) options enabled. The resulting CNV expression matrix was centered at 1. For each cell, a CNV score was calculated as the proportion of genes of which the inferred CNV values deviated from the expected range (0.9–1.1), defined as:

$$\:{CNV}_{score}=\:\frac{1}{N}{\sum\:}_{i=1}^{N}I({x}_{i}<0.9\:or\:{x}_{i}>1.1)$$

where *xi* is the inferred CNV value for gene *i*, *N* is the total number of genes analyzed, and *I* is the indicator function.

### Computational analysis of spatial transcriptomics data

To examine the spatial organization of functional programs associated with barcode groups, particularly the intratumoral distribution of persister subpopulations, spatial transcriptomics was performed on a BRAFi/MEKi-treated endpoint tumor using the 10x Genomics Visium platform. The expression matrix and spatial coordinates were processed using Seurat v5, and spots with > 20% reads mapped to the mouse genome were excluded.

As barcode information was not available in the Visium samples, each spot was annotated based on the functional signature with the highest AUCell enrichment score following normalization across all five signatures. For each annotated group, spatial autocorrelation was assessed using Moran’s Index statistic^31^. Cell-cell communication between groups was inferred using CellChat v2.1.2 [32, 33] in the spatial mode.

### Computational analysis of bulk RNA-seq data

Raw sequencing data were aligned to the human reference genome (hg38) using STAR v2.7.11b [34] and gene-level counts were quantified. Transcript abundance was normalized as transcript per million (TPM) for single-sample gene set enrichment analysis (ssGSEA) [35]. To assess temporal dynamics, the enrichment scores of the functional signatures identified in each barcode group were evaluated across time points.

### Analysis of acquired mutations

To assess the presence of acquired mutations following treatment, we applied the RNA_Mutect pipeline [36] to bulk RNAseq data. Whole-exome sequencing (WES) was performed on matched normal peripheral blood mononuclear cell (PBMC) samples.

Identified variants were functionally annotated using ANNOVAR [37], incorporating both the refGene and dbNSFP databases. This comprehensive annotation enabled the identification of potentially deleterious mutations by detecting variants absent in pre-treatment samples and recurrent across multiple post-treatment samples.

### Permutation-based analysis of recurrent gene- and pathway-level mutations

To test for genetically convergent resistance, we analyzed acquired somatic mutations called by RNA-MuTect from bulk RNA-seq of six independent endpoint tumors. For each tumor, the mutated gene set comprised unique genes with at least one post-treatment coding mutation absent from pre-treatment samples.

Gene-level recurrence was quantified as the number of tumors in which a given gene was mutated. Significance was assessed by permutation testing that preserved each tumor’s mutation burden. For each permutation, mutated gene sets were randomly sampled without replacement from a background universe of expressed genes while matching the number of mutated genes per tumor, and empirical p-values were calculated as the fraction of permutations with recurrence at least as high as observed.

Pathway-level recurrence was defined as the number of tumors in which at least one mutated gene mapped to a KEGG pathway. Permutations were performed analogously using the same burden-preserving randomized gene sets, with empirical p-values calculated as above and FDR estimated across pathways using Benjamini–Hochberg procedure.

### Alignment and analysis of mouse originated reads

To assess mouse stromal cell associated transcriptional signals in bulk RNA-seq data, sequencing reads were aligned to the mm39 mouse reference genome using STAR v2.7.11b, and gene-level counts were quantified and normalized to transcripts per million (TPM). For each time point, mean TPM values of curated marker gene sets for mouse fibroblasts, endothelial cells, pericytes, and macrophages were calculated. Average expression levels were visualized by bar plots to illustrate temporal changes in stromal transcriptional programs during treatment.

### Statistical tests

All statistical analyses were performed using R v4.4.0. Only post hoc false discovery rate (FDR) adjusted p-values less than 0.05 were considered significant.

## Results

### Development of MeRLin for integrated single-cell lineage tracing and transcriptomic profiling

**Fig. 1:**
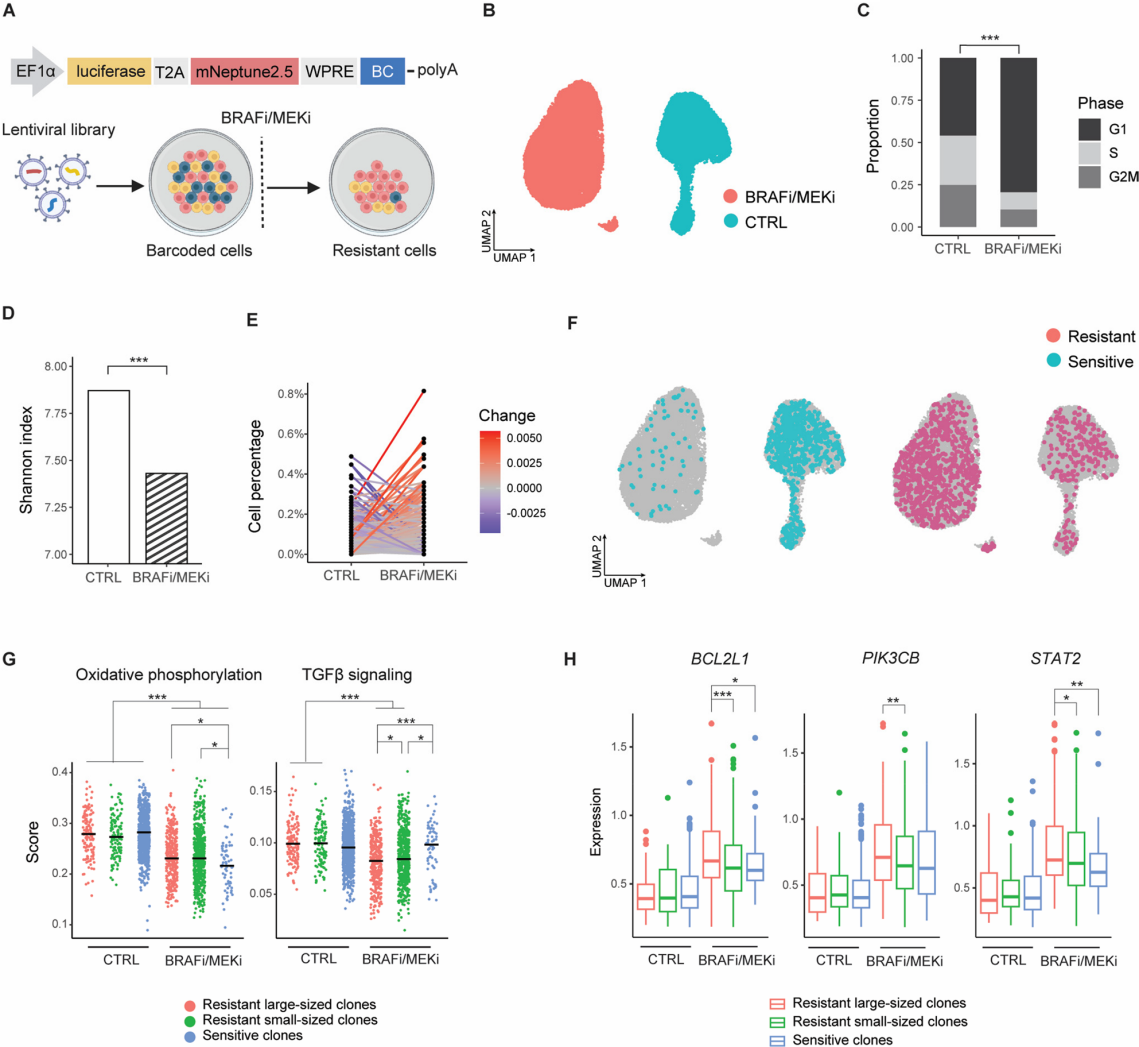
Distinct clonal fates are associated with variable transcriptional programs. (**A**) Schematic representation of the MeRLin construct and experimental workflow [41]. The vector encodes firefly luciferase and fluorescent protein mNeptune2.5. Lineage tracing is enabled by semi-random barcodes (BC) transcribed within the 3′ untranslated region of the mRNAs. WM4237-1 melanoma cells were transduced with the MeRLin barcode library. EF1α, elongation factor 1α; T2A, self-cleaving peptide; WPRE, woodchuck hepatitis virus post-transcriptional regulatory element; PolyA, polyadenylation signal. (**B**) Uniform manifold approximation and projection (UMAP) visualization of BRAFi/MEKi-treated (red) and control (CTRL, blue) WM4237-1 cells based on scRNA-seq data. (**C**) Distribution of cell cycle phases inferred from scRNA-seq. *P* < 2.2 × 10⁻¹⁶, two-tailed Fisher’s exact test. (**D**) Shannon diversity index comparing barcode diversity between CTRL and BRAFi/MEKi-treated cells. *P* < 2.2 × 10⁻¹⁶, two-tailed Hutcheson’s *t*-test. (**E**) Lineage tracing of BRAFi/MEKi-treated cells to their corresponding control cells (CTRL), quantified by the relative abundance of individual barcodes. (**F**) UMAP visualization highlighting sensitive (blue) and resistant (red) clones following BRAFi/MEKi treatment. (**G**) Differential expression analysis comparing resistant large-sized clones (> 15 cells), resistant small-sized clones (≤ 15 cells), and sensitive clones, revealing distinct transcriptional programs. (**H**) Statistical significance for differential expression analyses. * *P* < 0.05, ** *P* < 0.01, *** *P* < 0.001; two-tailed Wilcoxon rank-sum test

Phenotypic heterogeneity in cancer cell populations provides the foundation for clonal selection, particularly under drug pressure. Both Darwinian selection of pre-existing resistant clones and Lamarckian induction of drug-tolerant mechanisms can contribute to the development of DTP cells [1]. We developed MeRLin, a high-complexity lentiviral barcoding system designed to simultaneously track clonal dynamics and transcriptional states at single-cell resolution (Fig. 1A and S1A-S1C). The vector backbone encodes firefly luciferase and far-red fluorescent protein mNeptune2.5^19^, enabling in vivo imaging [18] (Methods and Fig. S1D). Lineage tracing is achieved by sequencing clone-specific transcribed barcodes within the 3′-untranslated region (UTR) of luciferase and mNeptune2.5 mRNAs (Methods). These barcodes consist of 265 bp semi-random sequences, allowing selected cancer subpopulations to be visualized using targeted RNA fluorescence in situ hybridization (RNA-FISH) (Table S1).

We constructed a MeRLin library of 2.89 million barcodes and transduced WM4237-1 melanoma cells with a multiplicity of infection (MOI) less than 0.4 to ensure each cell carried a unique barcode (Methods; Table S2). WM4237-1 was derived from a 28-year-old female patient with a BRAF V600E mutation prior to BRAFi/MEKi treatment [38] (Table S3). Four biopsies (WM4237-1 to -4) collected from the same patient at 2-4-month intervals shared identical mutation profiles (BRAFV600E, RB1N690fs, TP53S241), despite treatment with dabrafenib and trametinib combination therapy (best response: stable disease) administered between WM4237-1 and WM4237-2 [38]. Infected WM4237-1 cells were sorted for mNeptune2.5-positive populations using flow cytometry (Methods). The barcoded populations were expanded and divided into replicates, with one replicate saved as the original control.

### Resistant clones arise from minor populations present prior to treatment

We first analyzed the clonal selection of barcoded WM4237-1 cells under BRAFi/MEKi in vitro (Fig. 1A). Cell replicates were treated with 0.3 µM dabrafenib and 30 nM trametinib for a week, resulting in 20–30% survival of resistant cells (Methods). We performed scRNA-seq on both the BRAFi/MEKi-treated and control cells (Fig. 1B). Inferred cell cycle phase analysis revealed that treated cells exhibited G1 cell cycle arrest (Fig. 1C; Methods) and upregulation of melanocytic markers, including *EDNRB*,* DCT*, and *TYRP1* (Fig. S2A), indicating a more differentiated transcriptional state [39]. Single-cell regulatory network inference and clustering (SCENIC) analysis was employed to infer gene regulatory networks from the scRNA-seq data (Methods; Fig. S2B). *MYC* activity was higher in control cells, whereas *JUN* regulatory activity was increased in BRAFi/MEKi-treated cells (Fig. S2C).

Barcode reads were directly retrieved from scRNA-seq data, enabling lineage tracing of treated cells back to their corresponding origins in the vehicle control (Methods). This approach allowed simultaneous lineage tracing and direct comparison of transcriptional states between treated and control cells. Following BRAFi/MEKi treatment, barcode diversity was markedly reduced, with less than 25% of control cells surviving drug pressure (Fig. S2D). Consistently, the Shannon index, which measures barcode richness and evenness (Methods), showed a moderate reduction in barcode diversity in treated cells compared to controls (Fig. 1D). The dominant resistant clones originated from minor subpopulations present prior to treatment (Fig. 1E). Sensitive clones declined substantially following BRAFi/MEKi treatment (Fig. 1F, left and S2E), whereas resistant clones expanded (Fig. 1F, right and S2E). Because the most sensitive clones were completely eliminated by BRAFi/MEKi, only relatively sensitive clones remained detectable after treatment.

To identify the cellular expression programs associated with resistant clones, we analyzed differentially expressed gene sets in these cells (Methods). Resistant clones were separated into large-sized (> 15 cells) and small-sized (≤ 15 cells) groups using a published approach [4], enabling clearer distinction of expression patterns associated with different levels of drug resistance. Our analysis showed relative enrichment of oxidative phosphorylation (OXPHOS) in resistant clones (Fig. 1G). Although OXPHOS-related genes were downregulated in both sensitive and resistant clones following BRAFi/MEKi treatment, the reduction was most pronounced in sensitive clones. In the post-treatment setting, OXPHOS signatures remained enriched in both large- and small-sized resistant clones, distinguishing them from sensitive populations. In contrast, resistant clones exhibited marked downregulation of TGFβ (transforming growth factor β) pathway activity following BRAF/MEK inhibition, whereas sensitive clones retained active TGFβ signaling under the same conditions (Fig. 1G). Although expression of *BCL2L1*, *PIK3CB*, and *STAT2* increased across all treated cells compared to pre-treatment, these genes were expressed at higher levels in the most resistant clones compared with relatively sensitive cells (Fig. 1H). Notably, the corresponding control cells of the resistant clones largely lacked distinct transcriptional programs (Fig. 1F, right), suggesting that cancer cell heterogeneity may stem from stochastic and transient fluctuations in survival-associated genes and pathways [40].

### In vivo lineage tracing reveals clonal dynamics during MRD and recurrence

**Fig. 2:**
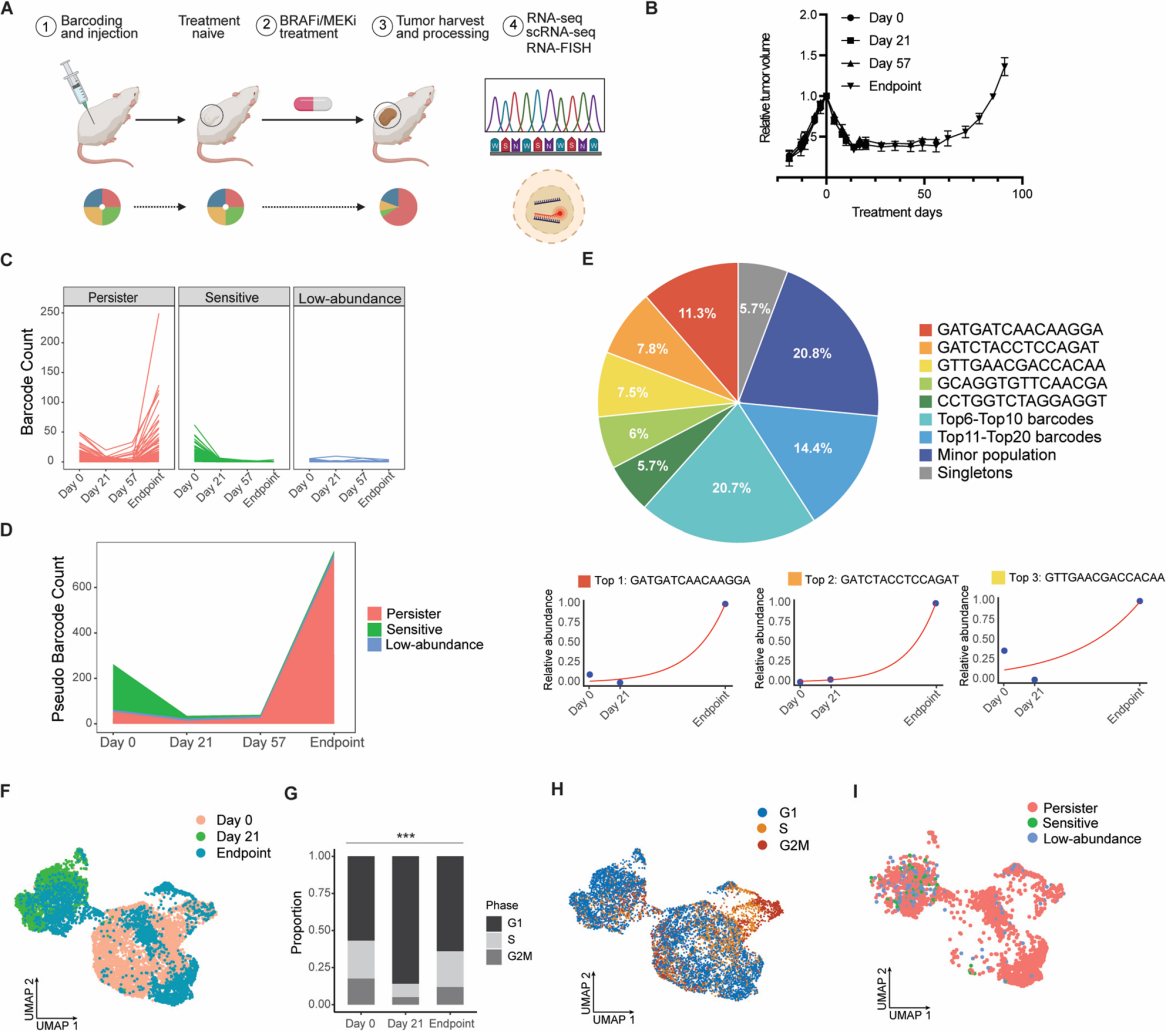
Clonal dynamics during BRAFi/MEKi treatment in a melanoma PDX model. (**A**) Experimental outline [41]. (1) MeRLin-barcoded WM4237-1 cells were injected subcutaneously into the right flanks of NSG mice. (2) BRAFi/MEKi treatment and tumor progression. (3) Tumors were harvested at multiple time points. (4) Transcribed barcodes were sequenced and visualized in situ by RNA-FISH. Pie charts illustrate changes in barcode composition during treatment. Tumor (mouse) numbers per time point: day 0 (pre-treatment), *n* = 3; day 21 (early MRD), *n* = 3; day 57 (late MRD or pre-recurrence), *n* = 3; endpoint (resistant), *n* = 6 (Table S4, bulk RNA-seq). (**B**) Growth curves of barcoded WM4237-1 tumors before and during BRAFi/MEKi treatment. (**C**) Normalized abundance of individual barcodes revealing distinct clonal fates, including persister subpopulations that initially responded to treatment but persisted and expanded during treatment (red), sensitive subpopulations that declined substantially following treatment (green), and low-abundance subpopulations that fluctuated without dominating resistant tumors (blue). (**D**) Stacked plot illustrating longitudinal clonal dynamics contributing to tumor recurrence during BRAFi/MEKi treatment. (**E**) Top, pie chart illustrating the hierarchical clonal composition of endpoint tumors based on scRNA-seq data, *n* = 3 (Table S4, scRNA-seq). The 15-bp barcode suffixes of the five most abundant clones are shown, with barcodes ranked 6–10, 11–20, minor populations, and singletons grouped accordingly. Bottom, normalized abundance of the top-ranked barcodes (rank 1–3) across prolonged treatment. (**F**) UMAP visualization of single-cell transcriptomes from pooled scRNA-seq data at day 0 (pre-treatment) (crimson red), day 21 (early MRD) (emerald green), and endpoint (resistant) (navy blue). (**G**) Proportion of cells in each inferred cell-cycle phase across time points. *** *P* < 0.001; two-tailed Fisher’s exact test. (**H**) UMAP colored by inferred cell-cycle phases, showing the distribution of G1 (royal blue), S (orange), and G2/M (brick red) cells. (**I**) UMAP visualization of endpoint tumor subpopulations classified as persister (red), sensitive (green), and low-abundance (blue) cells

We leveraged the patient-derived xenograft (PDX) model WM4237-1 [38] (Table S3), which recapitulates the patient’s response to BRAFi/MEKi therapy, initially showing strong sensitivity until relapse. Replicates of MeRLin-barcoded WM4237-1 cells were injected subcutaneously into the right flanks of immunodeficient NSG (NOD-*scid* IL2Rgamma^null^) mice and formed tumors (Fig. 2A; Methods). The barcoded tumors initially responded to BRAFi/MEKi treatment but relapsed after approximately three months (Fig. 2B; Methods).

We performed longitudinal analyses of WM4237-1 tumors collected at defined stages of treatment, including pre-treatment (day 0), early MRD (day 21), late MRD or pre-recurrence (day 57), and the resistant endpoint. Bulk RNA-seq was performed on the original control, three biological replicates each from day 0, day 21, and day 57, and six replicates from endpoint tumors (Fig. S3A). Barcode sequences extracted from bulk RNA-seq data were used to infer changes in clonal composition over time (Table S4; Methods). Approximately 22% of barcodes from pre-treatment tumors were detected in recurrent tumors following prolonged BRAFi/MEKi treatment (Fig. S3B). Consistent with this, Shannon diversity decreased at early MRD relative to pre-treatment and declined further by the pre-recurrence stage, reaching levels comparable to those observed in resistant tumors (Fig. S3C). Only six of approximately 200 barcodes were shared across all six recurrent tumor replicates (Fig. S3D), suggesting that tumor recurrence is unlikely to be driven predominantly by pre-existing resistant clones detectable in pre-treatment tumors [42].

We quantified the barcoded subpopulations by normalizing their proportions to the corresponding tumor volumes and global transcriptional activity across time points (Methods and Fig. S3F). Clonal dynamics were classified into three categories (Fig. 2C): (1) persister subpopulations, which initially responded to treatment but persisted and expanded during the MRD stage, ultimately dominating tumor recurrence; (2) sensitive subpopulations, which declined substantially following BRAFi/MEKi treatment; and (3) low-abundance subpopulations, characterized by low and fluctuating counts across time points, exhibited limited representation at tumor recurrence. The stacked plot illustrates the clonal dynamics of all three subpopulations, showing that dominant resistant clones primarily arose from minor subpopulations in pre-treatment tumors, whereas sensitive subpopulations declined substantially following treatment (Fig. 2D).

Among the six shared barcodes, three subpopulations belonged to persister cells (Fig. S3E), while the other three were classified as low-abundance subpopulations. Our analysis distinguished adaptive mechanisms from pre-existing resistance, supporting a predominant role for adaptive mechanisms in melanoma recurrence.

We applied RNA-MuTect to detect newly acquired somatic mutations in exons from bulk RNA-seq data of six resistant endpoint tumor replicates (Methods; Table S5). Although all tumors relapsed within approximately three months and each accumulated 200 to 300 genetic alterations in the coding regions, no recurrent mutations were shared across all samples (Fig. S3G). To assess whether mutation recurrence exceeded chance expectations, we performed permutation analyses at both gene and pathway levels [43] (Methods). At the gene level, although 9 genes including *SZT2*, were mutated in 4 of 6 replicates, recurrence was consistent with random chance (empirical *p* ≈ 0.13), with no mutated genes shared across 5 or 6 replicates. At the pathway level, we quantified recurrence as the number of endpoint replicates in which a KEGG pathway contained at least one mutated gene and applied permutation test that preserved each replicate’s mutation burden and sampled genes from the same background (Methods; Table S6). Although several pathways appeared recurrent in all replicates (*R* = 6/6), none remained significant after multiple-testing correction (minimum empirical *p* ≈ 6 × 10⁻⁴; FDR ≈ 0.11), including canonical cancer pathways such as MAPK and WNT signaling, indicating no pathway-level genetic convergence beyond chance.

### Single-cell analysis characterizes transcriptional states of persisters over time

We performed scRNA-seq on samples from treatment day 0, day 21, and the endpoint, pooling three replicate tumors per time point to characterize transcriptomic changes over time (Methods; Table S4). Since the sample preparation, sequencing workflow, and data processing differ between bulk RNA-seq and scRNA-seq, the absolute barcode read counts are only technically comparable within the same sequencing method. Barcode sequences extracted from scRNA-seq data were subjected to filtering steps including removal of UMI duplicates, empty vectors, short barcodes, barcodes with incorrect patterns, and cells containing multiple barcodes (Methods). To facilitate comparison with other barcoding technologies, we reported barcode performance metrics for both the in vitro and in vivo scRNA-seq datasets, including the average number of UMIs supporting each barcode call per cell (Fig. S4A-E), and the fraction of cells with a confident barcode assignment after quality control (Table S4).

Through scRNA-seq data, Shannon index similarly demonstrated a decrease in barcode diversity on day 21 compared to the pre-treatment tumor, and a further decline in the recurrent tumor (Fig. S4F). We detected 261 unique barcodes in the recurrent tumor, compared to 1,127 unique barcodes in the pre-treatment tumor, indicating that only 23% of the initial subpopulations survived prolonged BRAFi/MEKi therapy (Table S4). Resistant clones at the endpoint displayed a hierarchical distribution, with the most dominant clone comprising 11.3% of the cells from the three pooled tumors, and the top five persister lineages collectively accounting for 40% of the cells in recurrent tumors (Fig. 2E). The three top-ranked persister subpopulations underwent exponential growth during prolonged BRAFi/MEKi treatment (Fig. 2E; Methods).

Uniform manifold approximation and projection (UMAP) showed distinct transcriptional states between early MRD (day 21) and pre-treatment (day 0) tumors, whereas cells from the resistant (endpoint) tumors were located across both transcriptional states (Fig. 2F). The top three principal components (PC1-3) accounted for over 66% of the total transcriptomic variance (Table S7). PC1 was strongly enriched for cell cycle-associated genes (e.g., *MKI67*, *TOP2A*, and *BIRC5*), indicating that proliferative activity was a major factor distinguishing these longitudinal samples. Cell cycle phases inferred from scRNA-seq data demonstrated a higher proportion of cycling cells in G2/M and S phases in the populations of day 0 and endpoint tumors, while day 21 cells predominantly exhibited G1 cell cycle arrest (Fig. 2G; Methods). The corresponding UMAP illustrates how inferred cell-cycle phases contribute to transcriptional heterogeneity across time points (Fig. 2H). Barcode reads from single cells were quantified across time points using an approach similar to that applied to the bulk RNA-seq data, and cell populations were classified as persister, sensitive, or low-abundance subpopulations (Methods). Differential expression analysis comparing the low-abundance group with sensitive subpopulations did not identify any significant genes at any of the three time points using the specified thresholds (log2 fold-change ≥ 0.25 or ≤ − 0.25, cell percentage ≥ 10%, FDR < 0.05). UMAP visualization of endpoint tumor subpopulations defined by clonal fate showed that persister cells were preferentially distributed within transcriptional states characteristic of endpoint tumors (Fig. 2I and F). In contrast, inferred cell-cycle phase distributions did not differ significantly among persister, sensitive, and low-abundance subpopulations at the endpoint (Fig. S4G).

### Integrated lineage-transcriptome analysis identifies diverse persister programs

**Fig. 3:**
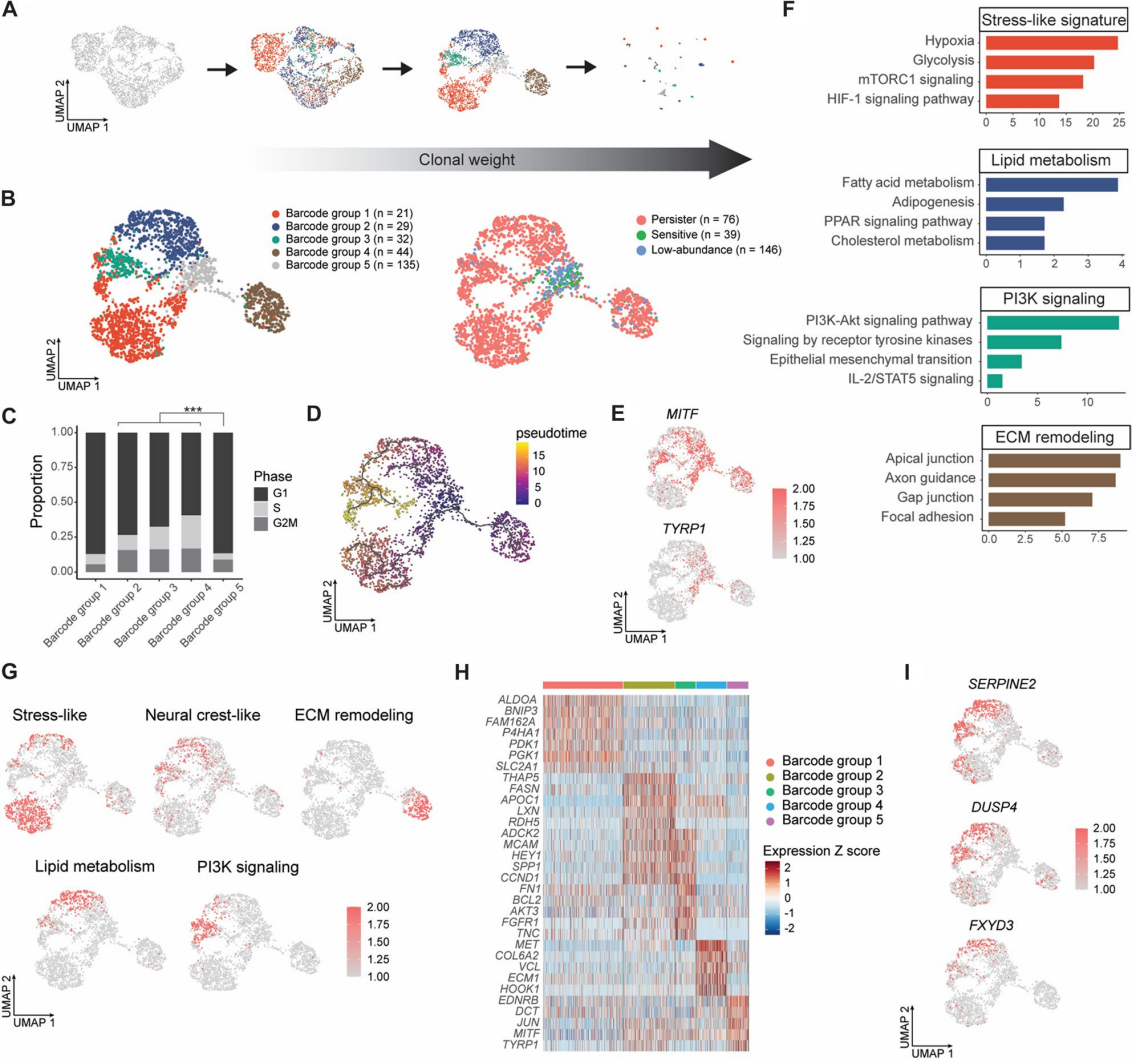
Distinct transcriptional programs characterize persister subpopulations. (**A**) ClonoCluster integration of clonal barcode identity (colored) with transcriptomic information (grey). UMAPs illustrate the effect of increasing Warp Factor values (0, 6, and 10) on the structure of endpoint tumor scRNA-seq data (Methods). (**B**) UMAP visualizations of hybrid clusters integrating clonal and transcriptomic information (left) and corresponding clonal fate assignments (right). Barcode groups 1–4 (red, blue, green, brown in the left panel) correspond to persister subpopulations (red in the right panel), whereas barcode group 5 (gray in the left panel) comprises sensitive (green in the right panel) and low-abundance (blue in the right panel) subpopulations. *n*, number of unique barcodes per group. (**C**) Proportion of cells in each inferred cell-cycle phase. *** *P* < 0.001; two-tailed Fisher’s exact test. (**D**) UMAP projection of single cells colored by inferred pseudotime values. Black curves indicate the inferred trajectory backbone. (**E**) UMAPs recolored by expression of the melanoma differentiation markers *MITF* and *TYRP1*. (**F**) Functional enrichment terms for barcode groups 1–4 identified in (**B**). *P*-values were determined using two-tailed Fisher’s exact tests (Table S10). (**G**) AUCell scores (color scale) for the top functionally enriched programs per barcode group projected onto the UMAP (Table S9). (**H**) Discriminative marker genes (5–7 genes per group) for each barcode group (Table S8, Endpoint). Fold change ≥ 2, cell percentage ≥ 50%, and FDR < 0.05. (**I**) UMAPs showing expression of *SERPINE2*, *DUSP4*, and *FXYD3* within barcoded subpopulations

We applied ClonoCluster [23], a clustering algorithm that integrates clonal identity and transcriptomic similarity, to generate hybrid clusters from scRNA-seq data independently at each time point (Fig. S5A-S5C; Methods). The resulting hybrid clusters were used to improve cluster resolution and facilitate marker identification [23]. By adjusting the contribution of clonal barcode identity within the UMAP embedding, ClonoCluster improved the separation of hybrid clusters (Fig. 3A; Methods). In the recurrent tumors, five barcode-defined groups were identified (Fig. 3B, left). Interestingly, barcode groups 1–4 corresponded to persister subpopulations defined by clonal fate (Fig. 3B, right), whereas barcode group 5 preferentially comprised sensitive and low-abundance subpopulations (Fig. 3B, right and S5D).

Examining cell cycle phases, we found that in recurrent tumors, persister subpopulations from groups 2 to 4 showed a significantly higher fraction of cycling cells in the G2/M and S phases, whereas sensitive and low-abundance subpopulations in group 5 predominantly underwent G1 cell cycle arrest (Fig. 3C; Methods). Pseudotime analysis revealed a continuous, branched transcriptional trajectory rather than a single linear progression, consistent with adaptive plasticity under treatment (Methods). The inferred trajectory indicated that barcode group 3 represented an extension of barcode group 2, whereas barcode groups 1 and 4 were preferentially enriched along distinct branches (Fig. 3D).

We detected a wide variability in the expression levels of *MITF*, a key regulator of melanoma differentiation [44], across all subpopulations in the recurrent tumor (Fig. 3E, top). Persister cells from group 1 exhibited the lowest *MITF* expression, consistent with a more dedifferentiated state relative to other groups. We also observed that *TYRP1*, a differentiation marker and *MITF* target gene, was highly expressed in the sensitive and low-abundance subpopulations of barcode group 5. (Fig. 3E, bottom). Similarly, additional differentiation markers including *DCT* and *EDNRB*, were enriched in these subpopulations (Fig. S6A).

To characterize the cellular expression programs underlying persister subpopulations, we identified differentially expressed genes in each barcode group (Methods; Table S8, Endpoint). EnrichR pathway analysis was used to functionally annotate each cluster (Methods; Table S9), revealing that group 1 was enriched for stress-like signatures [45], groups 2 and 3 shared neural crest stem-like (NC-like) features [45], and group 4 was associated with extracellular matrix (ECM) remodeling states (Fig. 3F and Table S10). The activities of these expression programs were measured using AUCell and projected onto UMAP space (Fig. 3G; Methods). The transcriptional states of groups 2 and 3 were associated with lipid metabolism and PI3K signaling, respectively [46] (Fig. 3F-G and Table S9).

Notably, the most dominant barcode (Fig. 2E, top 1) corresponded to the lipid metabolism state (Fig. S5E and 3G), barcodes ranked 2 to 5 mapped to the stress-like program (Fig. S5E and 3G), and the sixth-ranked barcode corresponded to the ECM remodeling state (Fig. S5E and 3G). To confirm representation of dominant barcodes in the pooled scRNA-seq dataset (Fig. 2E), we examined bulk RNA-seq-derived barcode frequencies for each of the three endpoint tumor replicates. Barcodes ranked 1–4 were found among the top five barcodes in at least one individual tumor replicate (Fig. S4H). Because bulk RNA-seq and scRNA-seq differ in sample processing, library preparation, and data analysis, barcode read counts are directly comparable within the same sequencing platform and not necessarily across platforms.

We performed CIBERSORTx deconvolution analysis of these persister states across 6 endpoint bulk RNA-seq replicates (Methods). These persister states were detected across nearly all tumor replicates (Table S11), suggesting that tumor recurrence is associated with convergence onto shared persister programs. Applying the same deconvolution analysis to longitudinal PDX samples WM4237-1 to WM4237-4, with BRAFi/MEKi therapy administered between WM4237-1 and WM4237-2, revealed a higher overall correlation of the four persister states in the post-treatment sample WM4237-2 than in the pre-treatment sample WM4237-1 (Table S11).

### Persister cells engage diverse programs to drive resistance

Persister cells from barcode group 1 exhibited a stress-like transcriptional program marked by increased expression of *BNIP3* and *PDK1*, together with elevated *ATF4*, *SLC2A1*, *ALDOA*, and *P4HA1* (Fig. 3H and S6B; Table S8, Endpoint). Cells from barcode groups 2 and 3 shared a transcriptional signature consistent with a neural crest stem-like state [45] (Fig. 3G), including expression of dedifferentiation-associated genes such as *MCAM* (CD146), *HEY1*, and *SPP1* (osteopontin) (Fig. 3H and S6C; Table S8, Endpoint). Although these NC-like subpopulations partially overlapped, they displayed distinct molecular features. Barcode group 2 showed preferential enrichment of lipid metabolism-related genes such as *FASN* and *APOE* (Fig. 3H and S6D), whereas barcode group 3 were enriched for PI3K signaling-associated markers, including *AKT3* and the receptor tyrosine kinase *FGFR1* (Fig. 3H and S6E; Table S8, Endpoint). Persister cells from barcode group 4 showed enrichment of genes involved in extracellular matrix (ECM) remodeling, including *ECM1*, *VCL* (vinculin), and *MET* (c-MET) (Fig. 3H and S6F; Table S8, Endpoint).

Furthermore, MeRLin-enabled transcriptomic profiling identified approximately 200 persister-enriched marker genes with significantly elevated expression across four persister subpopulations compared with sensitive and low-abundance subpopulations (Table S8, Endpoint, barcode groups 1–4). These persister subpopulations showed increased expression of *SERPINE2*, *DUSP4*, and *DUSP6* (Fig. 3I and S6G). In addition, we identified differentially expressed cell surface markers, including *MCAM* (CD146), *CSPG4*, *ITGA6*/*ITGA7*, and *FXYD3* (Fig. S6C and S6G), with *FXYD3* showing consistently increased expression across all persister groups (Fig. 3I).

### Distinct transcriptional regulators characterize persister states

**Fig. 4:**
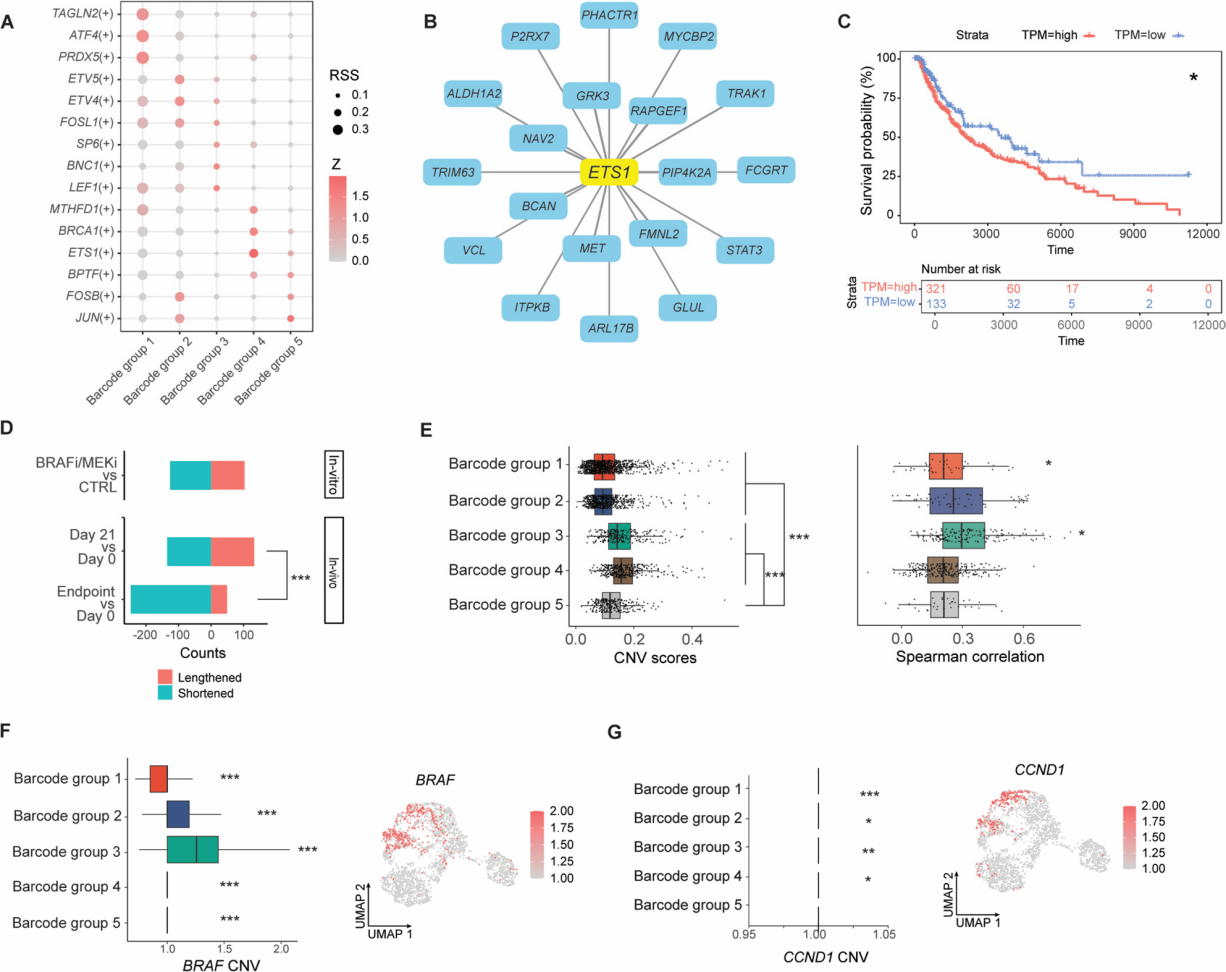
Regulatory features associated with adaptive resistance. (**A**) SCENIC analysis identifying transcriptional regulators enriched in each barcode group. (**B**) Regulon activity of *ETS1* and its predicted target genes in the ECM remodeling state. (**C**) Kaplan-Meier analysis of TCGA melanoma patients showing an association between higher *ETS1* expression and worse survival (*P* = 0.018). (**D**) Alternative polyadenylation analysis showing changes in 3′UTR length distributions across in vitro treatment and in vivo time points, compared to control cells and pre-treatment tumors. *** *P* < 0.001; two-tailed Fisher’s exact test. (**E**) Copy number variation scores across barcode groups shown on the left. *** *P* < 0.001; two-tailed *t*-test. Associations between CNV scores and gene expression within each barcode group are shown on the right using Spearman’s rank correlation coefficients. rs_1_ = 0.21, *P*_1_ = 0.047; rs_3_ = 0.28, *P*_3_ = 0.012 (median rs, subscripts indicate barcode groups). (**F**) Copy number variation of *BRAF* across barcode groups shown on the left. Spearman’s rank correlations between *BRAF* CNV and expression are shown for each barcode group, with UMAP visualization of *BRAF* expression shown on the right. rs_1_ = 0.44, rs_2_ = 0.56, rs_3_ = 0.65, rs_4_ = 0.23, rs_5_ = 0.29, (subscripts indicate barcode groups), *** *P* < 0.001. (**G**) Copy number variation of *CCND1* across barcode groups shown on the left. Associations between *CCND1* CNV and gene expression are shown using Spearman’s rank correlation coefficients, with UMAP visualization of *CCND1* expression shown on the right. rs_1_ = 0.23, rs_2_ = 0.1, rs_3_ = 0.17, rs_4_ = 0.13 (subscripts indicate barcode groups). * *P* < 0.05, ** *P* < 0.01, *** *P* < 0.001

SCENIC analysis revealed clear distinctions among stress-like, lipid metabolism, PI3K signaling, ECM remodeling, and melanocytic states (Methods; Fig. S6H). Specifically, *ATF4* was identified as the putative regulator of the stress-like state [47], *ETV5* for lipid metabolism [48], *LEF1* for PI3K signaling [49], and *JUN* for melanocytic state [50], respectively (Fig. 4A). Unsupervised analysis predicted *ETS1* as the transcription factor driving ECM remodeling in melanoma [51] (Table S12). A set of *ETS1* target genes were identified, including *VCL* and *MET* (Fig. 4B; Methods). Notably, five of the top ten genes most highly expressed in group 4 were *ETS1* targets, supporting a potential role for ETS1-regulated ECM remodeling programs in melanoma resistance. Higher *ETS1* expression was also associated with poorer patient survival in TCGA dataset (Fig. 4C).

### Post-transcriptional regulation and copy number variation in persister subpopulations

Next, we investigated the post-transcriptional mechanisms underlying adaptive resistance. scRNA-seq data were used for mining alternative polyadenylation (APA) isoform expression, which is a widespread regulatory mechanism that generates distinct 3′ transcript ends and is closely linked to cell identity, proliferation, and differentiation [52] (Methods). We observed both 3′UTR lengthening and shortening at early MRD (day 21) compared to pre-treatment (day 0) tumors, as well as in BRAFi/MEKi-treated melanoma cells compared with controls in vitro (Fig. 4D), indicating that alternative polyadenylation is actively modulated during early treatment response. However, with prolonged treatment, recurrent (endpoint) tumors showed globally shortened transcript isoforms, indicating that this shift may reflect the development of tumor resistance rather than a drug-induced effect observed at early MRD or in treated cells.

The copy number variation (CNV) content of each barcode group was ascertained from scRNA-seq data using inferCNV (Methods), indicating higher somatic copy number alterations in barcode groups 3 and 4 (Fig. 4E, left). In group 3, higher gene expression was significantly correlated with CNV, whereas no such correlation was observed in group 4 (Fig. 4E, right). Notably, increased *BRAF* expression was strongly associated with its copy number amplification in groups 2 and 3 (Fig. 4F), indicating that these persister subpopulations may sustain MAPK pathway activity through genomic amplification. In contrast, elevated expression of *CCND1* (cyclin D1), a key cell-cycle regulator, showed only a weak correlation with its CNV in groups 2 and 3 (Fig. 4G), suggesting that *CCND1* upregulation is likely driven by transcriptional or post-transcriptional mechanisms rather than genomic changes. To determine whether the inferred CNVs were clonal or subclonal, we reconstructed a heatmap of inferred copy-number variation profiles with genes ordered by genomic position and cells ordered by barcode groups (Fig. S6I). Large-scale CNV signals were continuous, highly consistent within individual barcode groups and were distinct across barcode groups, supporting a predominantly clonal, rather than subclonal origin of the inferred CNV patterns.

### Longitudinal clonal tracking reveals progressive diversification of persister programs

**Fig. 5:**
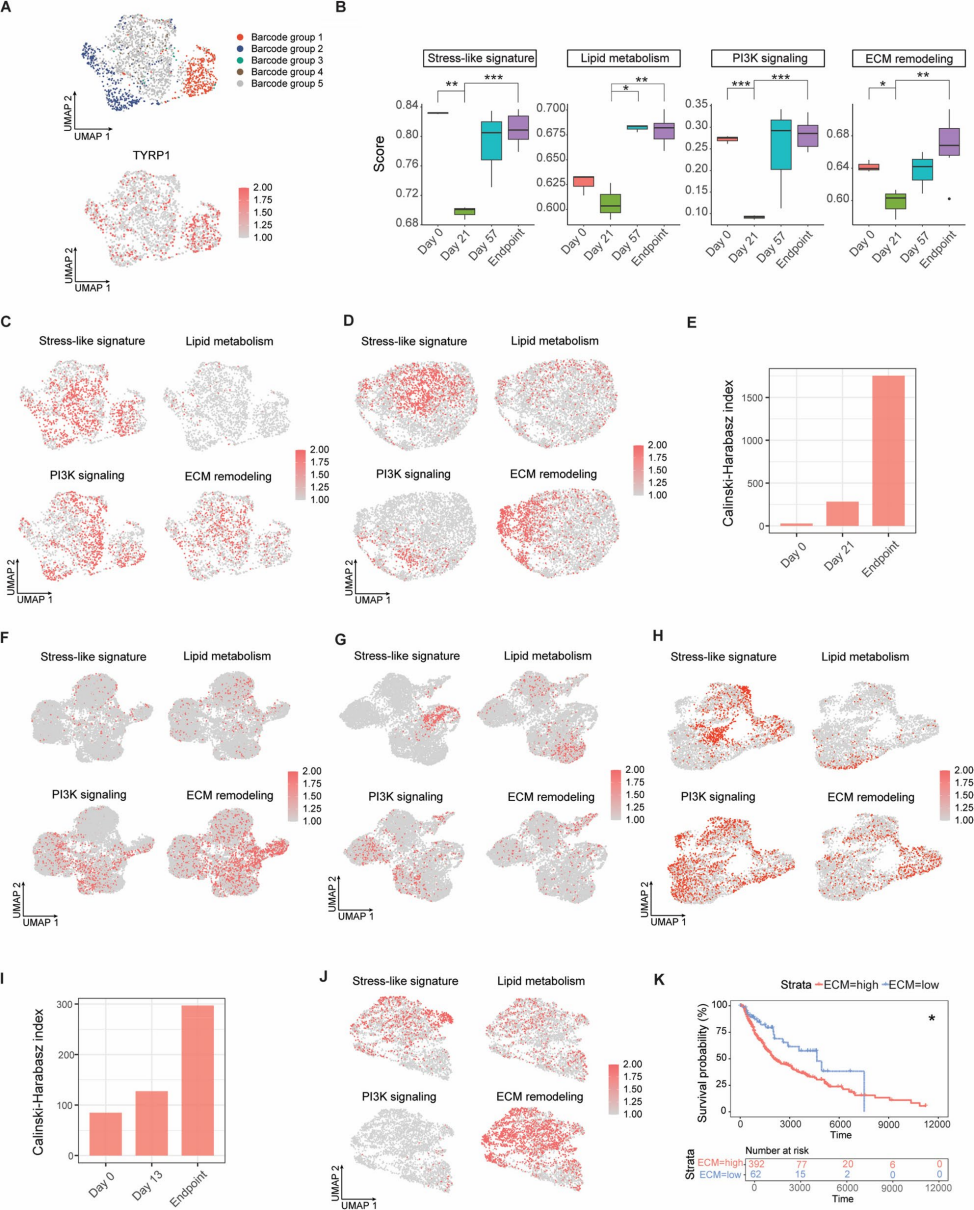
Persister programs are detected across treatment and PDX models. (**A**) ClonoCluster analysis applied to scRNA-seq data from early MRD (day 21) barcoded WM4237-1 tumors. UMAP showing projection of barcode groups 1–5, defined at the endpoint, onto the day 21 dataset (top). Expression of *TYRP1* is shown at day 21 and is enriched in barcode group 1 (bottom). (**B**) Pathway enrichment scores for stress-like, lipid metabolism, PI3K signaling, and ECM remodeling programs derived from bulk RNA-seq of barcoded WM4237-1 tumors across BRAFi/MEKi treatment. Sample numbers per time point are day 0 *n* = 3, day 21 *n* = 3, day 57 *n* = 3, and endpoint *n* = 6 (Table S4, bulk RNA-seq). * *P* < 0.05, ** *P* < 0.01, *** *P* < 0.001, two-tailed *t*-test. (**C-D**) UMAPs showing persister programs in barcoded WM4237-1 scRNA-seq data at early MRD (**C**) and pre-treatment (**D**). (**E**) Calinski-Harabasz index of persister programs across time points, quantified from ClonoCluster-derived UMAP embeddings of barcoded WM4237-1 scRNA-seq data. (**F-H**) UMAPs showing persister programs in non-barcoded WM4007 scRNA-seq data at tumor recurrence (**F**), early MRD (**G**) and pre-treatment (**H**). (**I**) Calinski-Harabasz index of persister programs across time points, quantified from UMAP embeddings of non-barcoded WM4007 scRNA-seq data. (**J**) UMAPs showing persister programs in scRNA-seq data from the non-barcoded, intrinsically resistant model WM4380-2. (**K**) Kaplan-Meier analysis of TCGA melanoma data showing that higher expression of the ECM remodeling signature is associated with poorer patient outcomes (*P* = 0.011)

Persister barcode groups identified in endpoint WM4237-1 tumors were longitudinally tracked by aligning exact barcode sequences to barcode reads detected at earlier time points, and transcriptional features of each barcode group were analyzed independently at each time point (Methods). Using ClonoCluster, which integrates clonal barcode identity with single-cell transcriptomic similarity, persister barcode groups 1 and 2 were detected at early MRD (day 21) (Figs. 3B and 5A). In contrast, barcode groups 3 and 4 were sparsely represented at this stage, consistent with the higher relative abundance of sensitive and low-abundance subpopulations during early MRD (Fig. S5F). Despite application of ClonoCluster, no transcriptionally distinguishable barcode groups were resolved at pre-treatment (day 0) (Fig. S7A and Fig. S5G), indicating limited clonal divergence prior to therapy.

At the early MRD stage, barcode group 1 exhibited 7 differentially expressed genes relative to other groups (Table S8, day 21), including upregulation of *TYRP1* (Fig. 5A), which is associated with a more differentiated transcriptional profile [39]. By contrast, at the endpoint, barcode group 1 showed the lowest *MITF* activity among all subpopulations (Fig. 3E), consistent with a more dedifferentiated state. Together, these observations indicate a phenotypic shift of barcode group 1 between early MRD and tumor recurrence, rather than a fixed persister identity established at early stages.

At early MRD, the 5 differentially expressed genes identified in barcode group 2 included *APOC1* (Table S8, day 21), which is associated with the lipid metabolism state (Table S9). Similarly, the 13 differentially expressed genes detected in barcode group 4 at early MRD included *MET* (Table S8, day 21), a gene associated with ECM remodeling (Table S9). These transcriptional features were weakly represented at early MRD and became more prominent at tumor recurrence. In contrast, no significantly differentially expressed genes were detected in barcode groups 3 or 5 at the early MRD stage.

Using bulk RNA-seq data, we profiled persister programs across multiple time points during BRAFi/MEKi treatment in the WM4237-1 model, including day 57 (Fig. 5B and Fig. S7B; Methods). At the bulk level, these programs were detected in pre-treatment tumors, showed reduced representation at early MRD (day 21), and exhibited higher representation at late MRD (day 57) and tumor recurrence. In parallel, bulk barcode analyses revealed changes in clonal composition between day 21 and day 57 (Fig. S3B and S3C), while overall tumor volumes remained relatively stable throughout the MRD period (Fig. 2B). Because bulk RNA-seq reflects aggregate expression across heterogeneous cell populations, these patterns indicate temporal changes in the overall representation of persister programs and clones without single-cell resolution.

To examine these programs in the scRNA-seq data at pre-treatment and early MRD, program activity was measured using AUCell and projected onto ClonoCluster-derived UMAP embeddings using the same approach applied for endpoint tumors (Fig. 3G; Methods). At early MRD, persister programs did not form separated clusters in the transcriptional embedding (Fig. 5C), including among barcode groups 1 and 2 that could be clonally defined at this stage (Fig. 5A), consistent with the limited number of differentially expressed genes between barcode groups at day 21 (Table S8, day 21). In pre-treatment tumors, these programs were also detected but were not clearly segregated either transcriptionally or by barcode group (Fig. 5D and Fig. S7A).

We quantified the separation of persister programs across time points using the Calinski-Harabasz (CH) index, which measures within-cluster coherence relative to between-cluster separation for a given embedding and clustering assignment. As shown in Fig. 5E, the CH index was lowest at pre-treatment, modestly higher at early MRD, and highest at tumor recurrence, indicating more clearly defined transcriptional patterns at recurrence. These analyses are consistent with increasing distinction of persister programs over the course of treatment (Table S8), while pre-treatment tumor cells may exhibit more variable and transient expression of resistance-associated genes and pathways without forming transcriptionally distinct clusters [40].

### Persister programs are present across PDX models

These persister programs were further evaluated in two independent, non-barcoded scRNA-seq datasets from BRAF V600E-mutant PDX models, including a BRAFi/MEKi-sensitive model WM4007 and a BRAFi/MEKi-resistant model WM4380-2 (Methods; Table S3). WM4007 initially responded to BRAFi/MEKi therapy but relapsed after approximately seven months [53] (Fig. S7C). Longitudinal samples from this model were collected at three stages, including pre-treatment (day 0), early MRD (day 13), and tumor recurrence (endpoint). scRNA-seq analysis showed that all four persister programs were detected at recurrence (Fig. 5F) and were also present at early MRD (Fig. 5G) and pre-treatment (Fig. 5H), although their transcriptional separation varied across time points. Quantification of program separation using the Calinski-Harabasz index showed higher values at later time points compared with pre-treatment (Fig. 5I), indicating greater transcriptional distinction of persister programs with prolonged treatment in the WM4007 model. Similarly, the Silhouette index, which assesses local cluster structure, increased over the course of treatment in both BRAFi/MEKi-sensitive models WM4237-1 and WM4007 (Fig. S7E and S7F).

WM4380-2 was derived from a stage IV melanoma patient previously treated with BRAFi [54], and showed no measurable response to BRAFi/MEKi treatment in the PDX model (Fig. S7D). All four persister states were present in this intrinsically resistant model, with particularly strong enrichment of the ECM remodeling program (Fig. 5J). Notably, higher expression of the ECM remodeling signature was associated with poorer patient outcomes in the TCGA melanoma cohort (Fig. 5K).

### Spatial mapping of persister states and cell-cell communication in recurrent melanoma

**Fig. 6:**
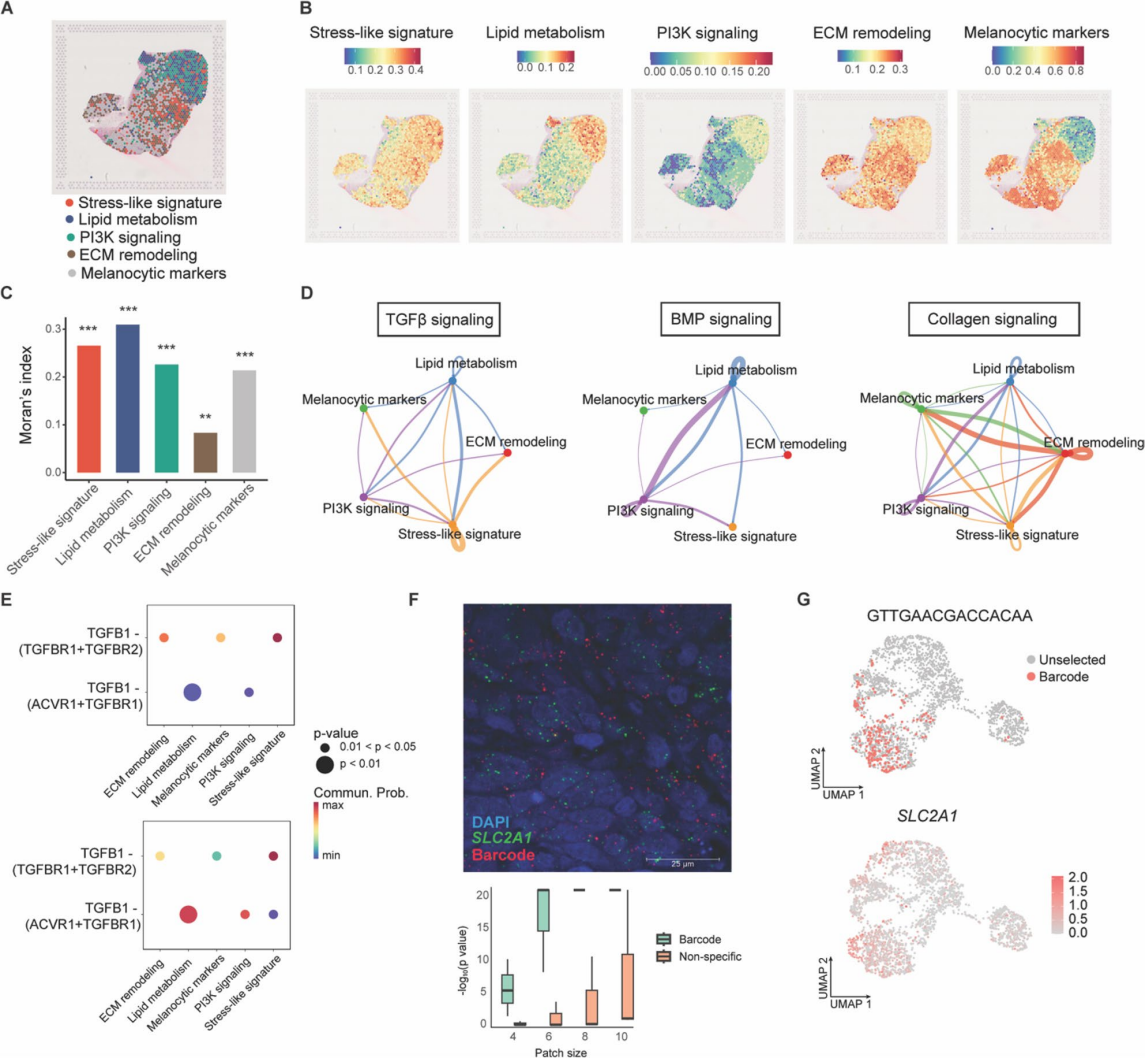
Spatial organization of persister states in recurrent melanoma. (**A**) Spatial transcriptomic annotation of persister states overlaid on a hematoxylin and eosin (H & E)-stained section of a recurrent WM4237-1 PDX tumor. Stress-like (red), lipid metabolism (blue), PI3K signaling (green), extracellular matrix (ECM) remodeling (brown), and melanocytic states (grey) are indicated. (**B**) Spatial distribution of individual persister states shown with normalized expression scales. (**C**) Moran’s index analysis quantifying spatial autocorrelation of each persister state. Stress-like, I = 0.27; lipid metabolism, I = 0.31; PI3K signaling, I = 0.23; ECM remodeling, I = 0.083; and melanocytic, I = 0.21. ** *P* < 0.01, *** *P* < 0.001. (**D**) Inferred cell-cell communication networks among persister states based on CellChat analysis, illustrating selected signaling pathways. Stress-like (yellow) and lipid metabolism (blue) states emitted TGFβ signals (left); lipid metabolism (blue) and PI3K signaling (purple) states exhibited autocrine and paracrine BMP signaling (middle); and ECM remodeling state (red) sent collagen signals (right). (**E**) Predicted ligand-receptor interactions underlying TGFβ signaling in (**D** left), showing outgoing signaling patterns from stress-like (top) and lipid metabolism states (bottom). (**F**) Barcode RNA-FISH demonstrating spatial localization of a dominant persister subpopulation (barcode suffix “GTTGAACGACCACAA”) and the stress-like marker *SLC2A1* in a recurrent tumor section (top). Statistical analysis (bottom) showing significant spatial co-localization (all patch sizes, Fisher’s exact test, *P* < 0.014), compared with a non-specific antisense barcode probe control. (patch size of 10 × 10, Fisher’s exact test, *P* = 0.089). The analysis was based on three ROIs (regions of interest) each. (**G**) UMAP projections showing expression of the dominant barcode and *SLC2A1*

To characterize the spatial organization of the persister states, we annotated stress-like, lipid metabolism, PI3K signaling, and ECM remodeling states using spatial transcriptomics (Methods). A published PDX dataset of WM4237-1 following prolonged BRAFi/MEKi treatment was analyzed to separate human transcriptomic content from mouse [53] (Fig. S8B), revealing the presence of these distinct transcriptional states in recurrent tumors (Fig. 6A and S8C). Stress-like signature showed a scattered distribution, whereas the ECM remodeling state formed a mosaic pattern interspersed with melanocytic cells (Fig. 6B and S8D). Notably, lipid metabolism and PI3K signaling signatures associated with the NC-like state were co-localized and enriched near the tumor boundary (Fig. 6B). Moran’s index revealed that the ECM remodeling state exhibited the weakest spatial autocorrelation among the signatures, indicating a relatively low degree of spatial dependence (Fig. 6C and S8E; Methods).

We used CellChat to quantitatively infer cell-cell communication networks among persister states (Methods). The outgoing signaling of TGFβ and Midkine (*MK*) was primarily observed in stress-like cells [55] (Fig. 6D and S9A). Predicted cell-cell communication between lipid metabolism and PI3K signaling states included BMP (bone morphogenetic protein), ANGPTL (angiopoietin-like protein), CSPG4 (chondroitin sulfate proteoglycan 4), and MPZ (myelin protein zero, P0), all of which are associated with neural differentiation [56] (Fig. 6D and S9B). Notably, *MPZ* was upregulated in lipid metabolism associated barcode group 2 (Table S8, Endpoint). Collagen and claudin (CLDN) signaling was strongly upregulated in the ECM remodeling state (Fig. 6D and S9C). Additional representative signaling pathways among persister states are shown in Fig. S9D-F. We characterized the specific ligand-receptor pairs involved in TGFβ1 signaling and found that the outgoing signaling pattern of stress-like cells primarily involved TGFβR1 (ALK5) and TGFβR2, whereas the lipid metabolism state showed outgoing signaling through either TGFβR2 or ACVR1 in combination with TGFβR1 (Fig. 6E).

### MeRLin enables in situ profiling of clonal identity and gene expression.

To demonstrate our barcode RNA-FISH strategy as a proof of principle for in situ profiling of clonal identity and gene expression, we recovered the full-length barcode sequence of a selected subpopulation from bulk RNA-seq data (Methods). RNA-FISH probes were designed to target the barcode suffix “GTTGAACGACCACAA”, which corresponds to the rank 3 subpopulation (Figs. 2E and 7.5% of the recurrent tumor) and maps to the stress-like state of barcode group 1 (Fig. S5E and 3G). Using standard RNA-FISH (Methods), we spatially mapped this dominant persister subpopulation and quantified its co-occurrence with the stress-like marker *SLC2A1* (Fig. 6F, top). Statistical analysis revealed a significant colocalization between the dominant barcode signal and *SLC2A1* expression in the recurrent tumor (Fig. 6F, bottom). In contrast, RNA-FISH probes targeting the antisense sequence of the same barcode, used as a negative control for non-specific staining, showed no colocalization (Methods; Fig. 6F, bottom). Consistent with the scRNA-seq results, *SLC2A1* was upregulated in the selected persister subpopulation (Fig. 6G), supporting its association with the stress-like transcriptional program of barcode group 1 observed in recurrent tumors (Fig. 3B and G). Together with the computational framework for identifying representative markers of persister subpopulations, this proof-of-principle experiment demonstrates that our barcode RNA-FISH strategy enables in situ clonal profiling and transcript quantification at single-cell resolution.

## Discussion

Under sustained targeted therapy, melanoma cells can undergo adaptive reprogramming rather than merely enduring drug pressure [3]. Drug-tolerant persister (DTP) cells represent key survivors that reshape signaling pathways and cellular identities to evade therapeutic inhibition [45]. However, the temporal dynamics and clonal context of these adaptive changes remain incompletely understood. To address this challenge, we developed MeRLin, a lineage tracing platform that integrates cellular barcoding, single-cell transcriptomics, and RNA-FISH to characterize clonal dynamics together with transcriptional plasticity and spatial organization during melanoma treatment [42].

Using MeRLin in WM4237-1 melanoma cells treated in vitro with BRAFi/MEKi, we found that resistant clones exhibited relative enrichment of OXPHOS activity and higher expression of *BCL2L1*, *PIK3CB*, and *STAT2* compared with sensitive clones. Since these genes were selectively upregulated in resistant clones after treatment, functional studies will be needed to determine whether targeting these genes and pathways can sensitize resistant cells to BRAFi/MEKi. Resistant clones also showed reduced TGFβ pathway activity after treatment, whereas sensitive clones retained active TGFβ signaling, consistent with prior evidence that TGFβ can promote apoptosis when combined with MAPK pathway inhibition [57]. These data suggested that attenuation of TGFβ signaling may represent a mechanism to evade pro-apoptotic responses, although its contribution to drug resistance remains to be validated.

Application of MeRLin to PDX model WM4237-1 revealed that therapeutic resistance arose predominantly through adaptive reprogramming rather than selection of pre-existing resistant clones. Dominant resistant clones in recurrent tumors originated from initially minor subpopulations that were transcriptionally similar to other groups prior to treatment and progressively acquired resistance-associated features over time. Although we cannot exclude the presence of rare pre-existing resistant clones below our detection threshold, the lack of gene- and pathway-level genetic convergence across endpoint tumors supports a model in which phenotypic plasticity is a major contributor to melanoma recurrence.

Integrated clonal and transcriptomic analyses identified four persister-associated states characterized by stress-like, lipid metabolism, PI3K signaling, and extracellular matrix (ECM) remodeling programs, suggesting that melanoma persister cells can access multiple adaptive routes [58]. The stress-like state of barcode group 1 was marked by increased expression of *PDK1*, *SLC2A1*, and *ALDOA*, consistent with a glycolytic shift previously associated with the Warburg effect [7], together with upregulation of hypoxia-associated genes *BNIP3* and *P4HA1* linked to melanoma progression [7], and activation of *ATF4*-mediated stress signaling implicated in escape from BRAF inhibition [47]. Neural crest-like signature in barcode groups 2 and 3 was consistent with established models of melanoma dedifferentiation [45]. The lipid metabolism state was characterized by *FASN* and *APOE*, genes previously implicated in MAPK inhibitor resistance through reducing lipid poly-unsaturation and protecting of *MITF*-low/*AXL*-high persister cells from ferroptosis [59, 60]. The PI3K signaling state featured *AKT3* and *FGFR1*, consistent with their roles in apoptosis resistance and therapy tolerance [61, 62]. The ECM remodeling state of barcode group 4 showed upregulation of *ECM1*, *VCL*, and *MET*, linking extracellular matrix dynamics to epithelial-mesenchymal transition [63], mechanosignaling and cancer progression [64]. Longitudinal clonal tracking further revealed that the stress-like persister state underwent a pronounced transition from a relatively differentiated phenotype at early MRD to a more dedifferentiated state at recurrence, whereas phenotypic features of the lipid metabolism and ECM remodeling states were weakly evident at early MRD and progressively consolidated during tumor recurrence.

Post-transcriptional regulation was also associated with adaptive resistance, with dynamic 3′UTR lengthening and shortening during early MRD and in vitro treatment, followed by global 3′UTR shortening in recurrent tumors. This pattern is consistent with a shift in alternative polyadenylation from a drug-induced MRD state toward a more proliferative state at recurrence, potentially through reduced miRNA-mediated repression [65]. In parallel, CNV analyses suggested that barcode groups 2 and 3 combined copy number amplification reinforcing MAPK signaling, such as *BRAF* amplification, with non-genetic adaptations that promote cell-cycle re-entry, including increased *CCND1* expression, highlighting the coexistence of genetic and non-genetic mechanisms. We also identified cell surface markers enriched in DTP populations, including *FXYD3*, which may serve as candidate biomarkers, although their functional relevance remains to be determined.

Deconvolution of bulk RNA-seq data indicated that the four persister programs were consistently detected across endpoint tumor replicates and showed greater concordance in the post-treatment PDX sample WM4237-2 than pre-treatment PDX. These programs were not unique to recurrence but were present across models and treatment stages, including two BRAFi/MEKi-sensitive models WM4237-1 and WM4007, as well as an intrinsically resistant model WM4380-2. In BRAFi/MEKi-sensitive models, we found that the transcriptional distinction of these programs increased under prolonged therapy. Nonetheless, these persister programs should be interpreted with appropriate caution, as some transcriptomic features may be influenced by replicate-specific factors including clonal CNVs, and additional biological replicates, extended time points, or alternative models may reveal additional persister programs or further heterogeneity.

Among the persister states, ECM remodeling-associated barcode group 4 represented a distinct subpopulation. These cells displayed pronounced transcriptional separation, enrichment along an independent pseudotime branch, and the highest proliferative capacity at recurrence. SCENIC analysis identified *ETS1* as a candidate regulator of this program [51], and higher *ETS1* expression together with higher ECM remodeling signature were associated with poorer patient outcomes in TCGA melanoma cohorts. Spatial analyses further showed that ECM remodeling cells formed a dispersed mosaic pattern interspersed with melanocytic cells, consistent with a dynamic and potentially advanced adaptive state. While these associations support an important role for ECM remodeling in melanoma resistance, functional studies will be required to establish causality.

Although the WM4237-1 model was analyzed in both in vivo and in vitro settings, notable differences were observed. Extensive 3′UTR shortening observed in recurrent tumors in vivo was not detected in BRAFi/MEKi-treated cells in vitro or at the MRD stage, whereas melanocytic genes were upregulated in resistant clones in vitro but suppressed in persister populations at the endpoint in vivo. These findings suggest that in vitro systems may primarily capture immediate treatment responses, whereas resistance-associated programs develop under in vivo microenvironmental influences, including stromal interactions, hypoxia, and spatial constraints [17]. Because PDX models more closely recapitulate human melanoma biology, they may offer greater translational relevance than in vitro systems [16]. As our scRNA-seq analyses focused on human melanoma cells, future studies incorporating longitudinal profiling of stromal and immune cells, ideally in humanized mouse models, will be important for defining tumor microenvironment contributions to DTP development.

Spatial transcriptomic analysis revealed a structured organization of persister states, with lipid metabolism and PI3K signaling states co-localized near the tumor boundary, while ECM remodeling cells showed minimal spatial dependence. Inferred cell-cell communication analyses suggested coordinated autocrine and paracrine signaling among persister states, in which stress-like cells secrete TGFβ, which has been reported to promote fibronectin deposition and establishment of a resistant niche [66], while ECM remodeling cells engage collagen and claudin (*CLDN*) pathways associated with a shift toward a differentiated, proliferative phenotype [67]. In addition, MeRLin-enabled barcode RNA-FISH provided proof-of-principle validation for in situ clonal profiling, demonstrating spatial co-localization of a dominant persister subpopulation with the stress-like marker *SLC2A1*.

Prior lineage tracing approaches include static barcoding systems such as LARRY, CellTag, and Watermelon for scRNA-seq based clonal labeling, FateMap combined with ClampFISH for spatial validation, and imaging-based lineage recorders such as intMEMOIR, baseMEMOIR, and PEtracer that use sequential rounds of FISH-based hybridization to read out high-complexity evolving barcodes. Although MeRLin requires custom RNA-FISH probes that are designed after barcode identification by sequencing, which limits spatial throughput, its spatial readout is intended as a targeted spatial validation strategy rather than comprehensive clonal mapping and was therefore applied selectively in this study.

In summary, this study provides a multidimensional view of tumor heterogeneity and clarifies the relationship between clonal dynamics and phenotypic plasticity during melanoma therapy resistance [4, 7, 11]. By integrating clonal tracking with transcriptional profiling and spatial context at single-cell resolution, MeRLin reveals how adaptive reprogramming shapes persister clonal dynamics and highlights candidate programs and genes that warrant further functional investigation [1].

## Supplementary Information

Supplementary Material 1.

Supplementary Material 2.

## Data Availability

Raw and processed scRNA-seq and bulk RNA-seq data were deposited in the Gene Expression Omnibus (GEO) under the accession number GSE299711 and GSE299589. Raw and processed Visium spatial transcriptomic data are available under accession number GSE245582. Raw scRNA-seq data for the WM4380-2 model can be requested from Dr. Vito W. Rebecca’s preprint paper [54]. The analysis and visualization scripts for this study are available at GitHub (https://github.com/Yeqing95/MeRLin), along with a brief guide on reproducing the analysis workflows and figures presented in the paper.
