# Supplementary material for: Clonal dynamics shaped by diverse drug-tolerant persister states in melanoma resistance
Source: Mol Cancer. 2026 Mar 3;25:127. doi: 10.1186/s12943-026-02622-9 (PMC13162433; doi:10.1186/s12943-026-02622-9)
Supplement: Supplementary file 1 — Supplementary Material 1. [file 12943_2026_2622_MOESM1_ESM.docx]

**Supplementary Figures**

**
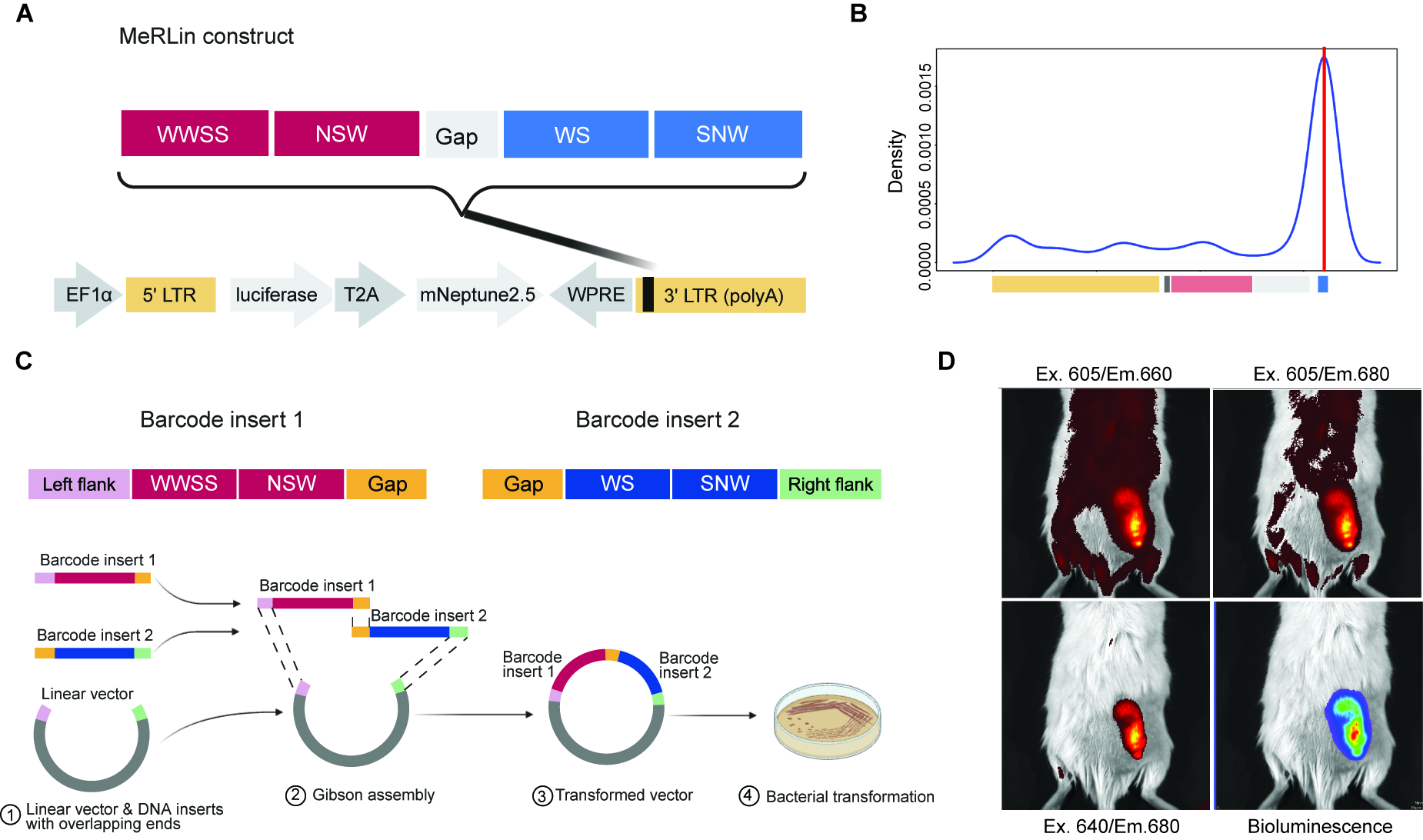
**

**Fig. S1 MeRLin vector design and validation for barcode detection by scRNA-seq, RNA-FISH, and in vivo imaging.**

**(A)** Schematic of the MeRLin vector design^13^. The vector encodes firefly luciferase and the far-red fluorescent protein mNeptune2.5, along with semi-random barcodes transcribed in the 3′ untranslated region (Methods). EF1α, elongation factor 1α; LTR, long terminal repeat; PolyA, polyadenylation signal; WPRE, woodchuck hepatitis virus post-transcriptional regulatory element. WWSS, NSW, WS, and SNW sequences are each repeated for 60 nucleotides (W = A or T; S = G or C; N = any base); Gap, sequence bridging the two barcode inserts (Table S1).

**(B)** Coverage plot of scRNA-seq data showing the optimal barcode position within the vector for direct retrieval of barcode reads. Lentiviral components are color coded at the bottom, with luciferase shown in yellow, mNeptune2.5 in red, and barcodes in blue.

**(C)** Schematic of MeRLin plasmid library construction using Gibson assembly^13^ (Methods and Table S1). are compatible with targeted RNA fluorescence in situ hybridization (RNA-FISH) for visualization of selected cancer subpopulations (Methods).

**(D)** Representative in vivo imaging using the IVIS Spectrum system of a mouse bearing a MeRLin-barcoded WM4237-1 tumor, showing detection by bioluminescence and mNeptune2.5 fluorescence under far-red excitation and emission settings optimized for tissue penetration.


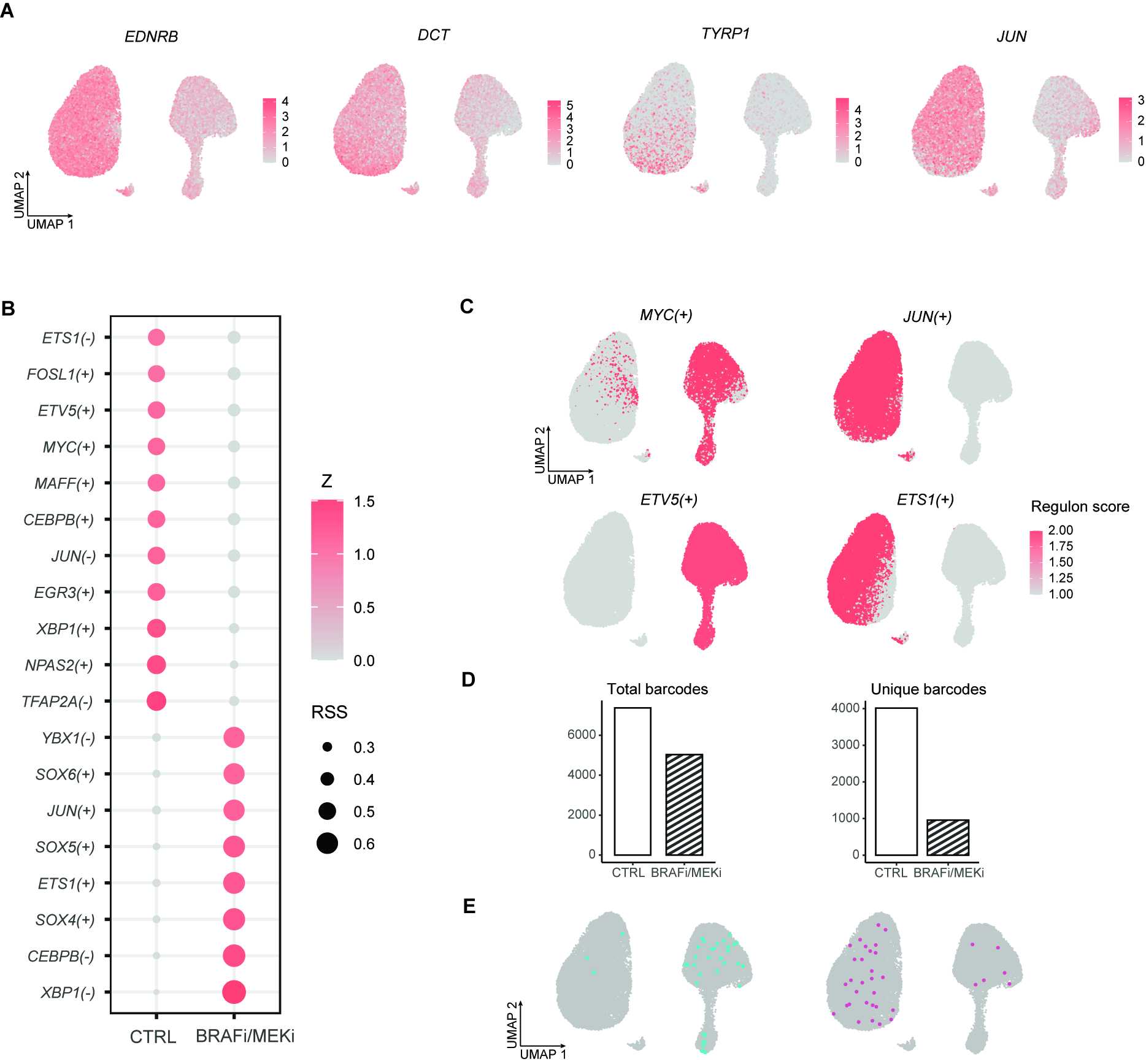


**Fig. S2 Transcriptional and clonal changes in WM4237-1 cells following BRAFi/MEKi treatment.**
**(A)** UMAP visualization showing expression of melanocytic genes EDNRB, DCT, and TYRP1, together with the transcription factor JUN, across control and BRAFi/MEKi-treated WM4237-1 cells.
**(B)** SCENIC analysis of scRNA-seq data highlighting transcription factors with enriched regulon activity in treated cells.

**(C)** Regulon activity scores for transcription factors MYC, JUN, ETV5, and ETS1 displayed across single cells.

**(D)** Total barcode counts and numbers of unique barcodes detected following BRAFi/MEKi treatment.
**(E)** UMAP visualization showing one representative sensitive clone (blue) and one representative resistant clone (red) based on barcode identity.


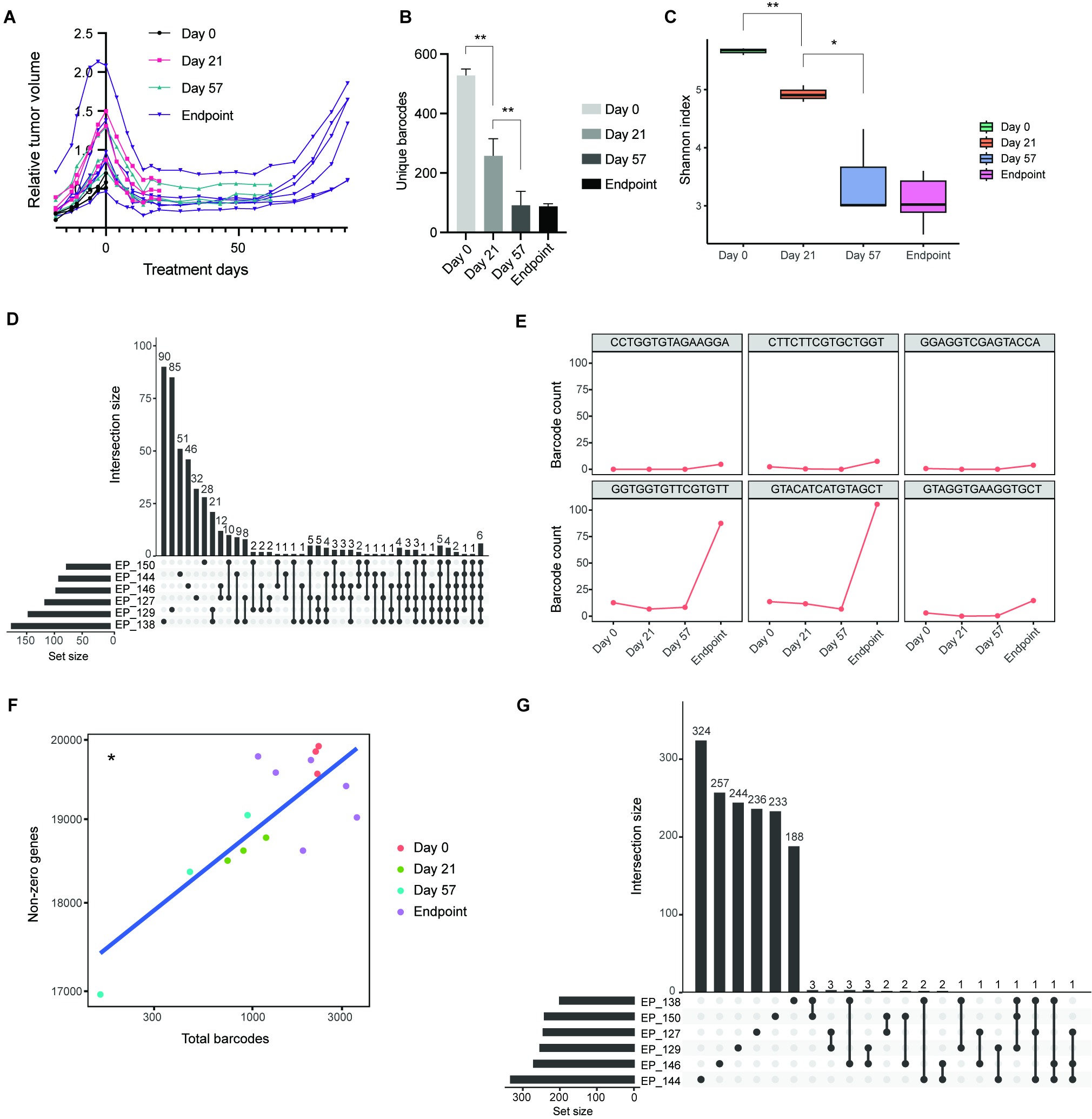


**Fig. S3 Barcode distribution across BRAFi/MEKi-treated WM4237-1 tumors based on bulk RNA-seq data.**

**(A)** Growth curves of individual MeRLin-barcoded WM4237-1 tumors during BRAFi/MEKi treatment. Tumor harvest time points are indicated by four color groups.

**(B)** Number of unique barcodes detected at each treatment time point based on bulk RNA-seq data. ** P < 0.01, one-tailed t-test.

**(C)** Shannon diversity indices showing changes in barcode diversity over time, with a moderate decrease at day 21 and a further reduction at day 57. * P < 0.05 and ** P < 0.01, one-tailed t-test.
**(D)** Plot showing barcodes shared among endpoint (EP) replicate tumors.

**(E)** Normalized abundance of the 6 shared barcodes identified in panel (**D**) across treatment time points.
**(F)** Scatter plot showing global transcriptional activity across time points for genes and barcodes with non-zero expression. Pearson correlation coefficient r = 0.58, P = 0.025.

**(G)** RNA-MuTect analysis showing no shared acquired mutations among the 6 endpoint (EP) tumors relative to pre-treatment tumors.

**
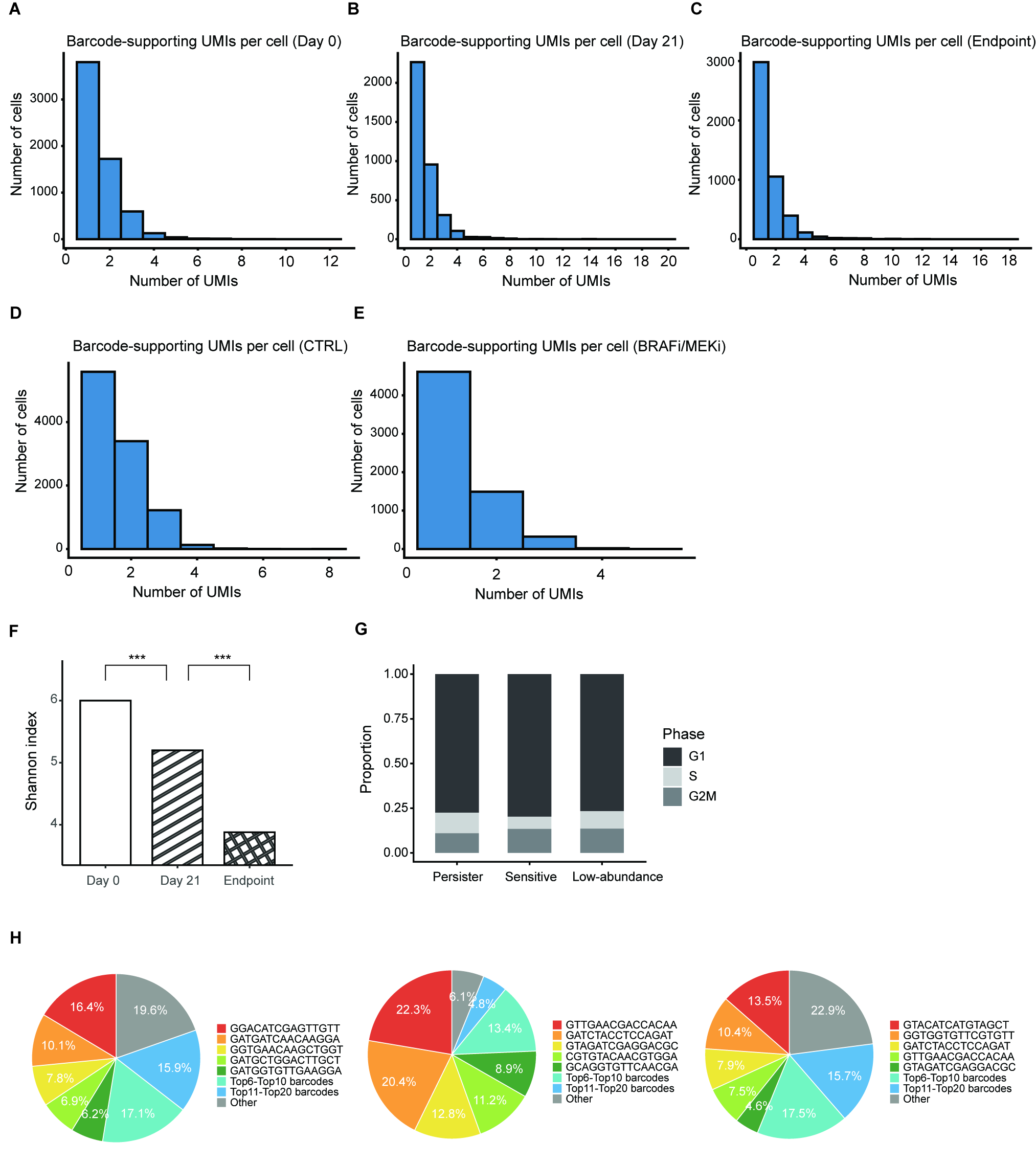
**

**Fig. S4 Clonal representation in BRAFi/MEKi-treated WM4237-1 cells and tumors assessed by scRNA-seq.**

**(A-C)** Histograms showing the number of barcode-supporting UMIs per cell in the *in vivo* WM4237-1 scRNA-seq dataset at day 0 (**A**), day 21 (**B**), and endpoint (**C**). The x-axis denotes the number of barcode-supporting UMIs per cell and the y-axis denotes the number of cells.

**(D-E)** Histograms showing the number of barcode-supporting UMIs per cell in the *in vitro* scRNA-seq datasets under vehicle control (CTRL) (**D**) and BRAFi/MEKi treatment (**E**).

**(F)** Shannon diversity indices showing changes in barcode diversity over time, with a decrease observed by day 21 and a further reduction at the resistant endpoint. *** *P* < 0.001, two-tailed Hutcheson *t*-test.

**(G)** Proportion of cells in each inferred cell-cycle phase across persister, sensitive, and low-abundance subpopulations within endpoint tumors.

**(H)** Pie charts showing the relative abundance of dominant barcodes in each of the three endpoint tumor replicates that were pooled for scRNA-seq, based on barcode frequencies measured by bulk RNA-seq.

**
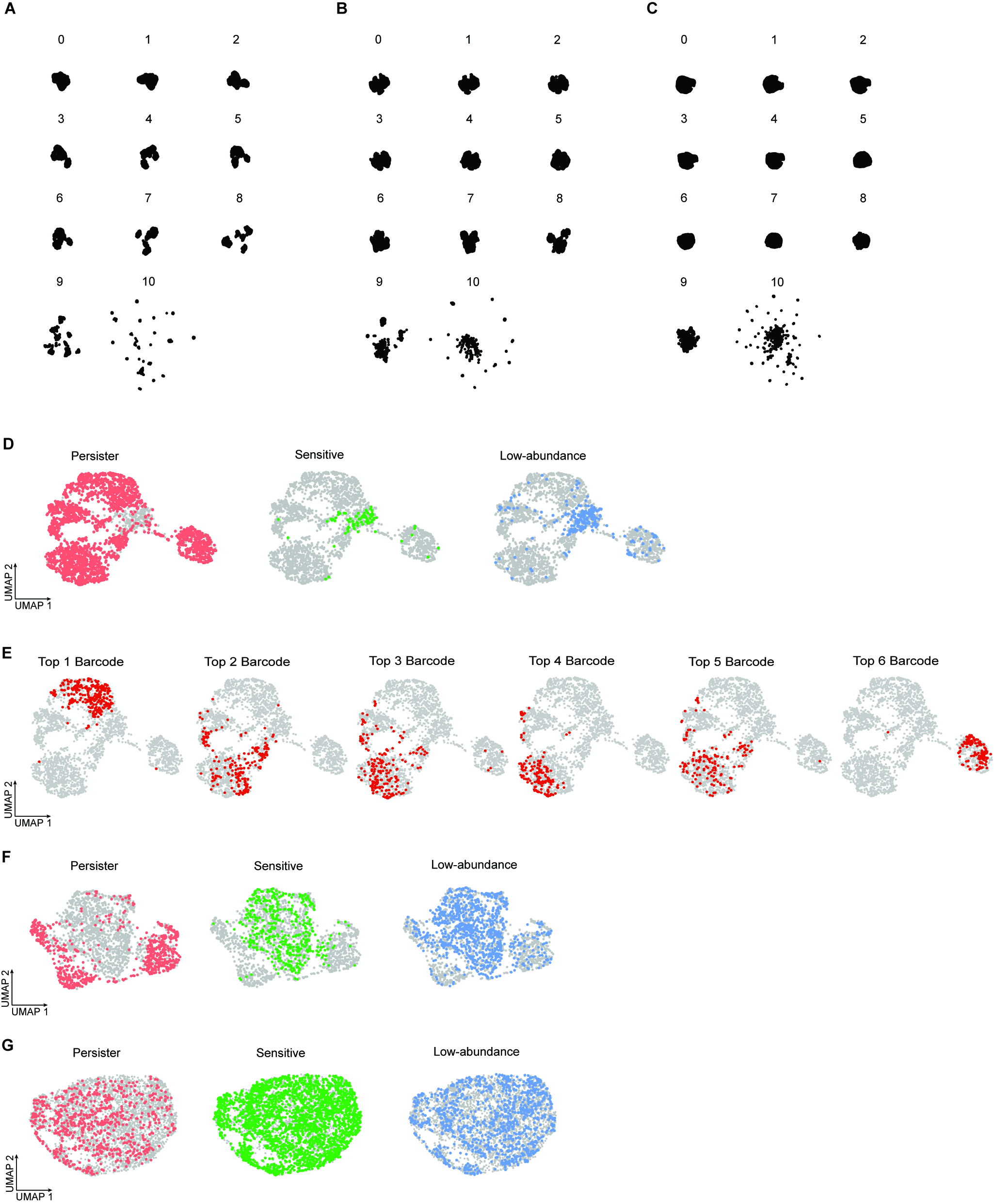
**

**Fig. S5 ClonoCluster integration of clonal and transcriptomic information in WM4237-1 tumors.**
**(A-C)** Effect of increasing Warp Factor values on UMAP structure for scRNA-seq data from WM4237-1 endpoint tumors (**A**), day 21 tumors (**B**), and day 0 tumors (**C**).

**(D)** UMAP visualizations of hybrid clusters generated by ClonoCluster, with cells categorized as persister (red), sensitive (green), or low-abundance (blue) based on clonal fates.

**(E)** UMAPs showing the expression patterns of the most abundant barcodes ranked 1-6 in Fig. 2E.

**(F)** UMAP visualizations showing persister (red), sensitive (green), and low-abundance (blue) subpopulations in WM4237-1 day 21 tumors.

**(G)** UMAP visualizations showing persister (red), sensitive (green), and low-abundance (blue) subpopulations in WM4237-1 day 0 tumors.

**
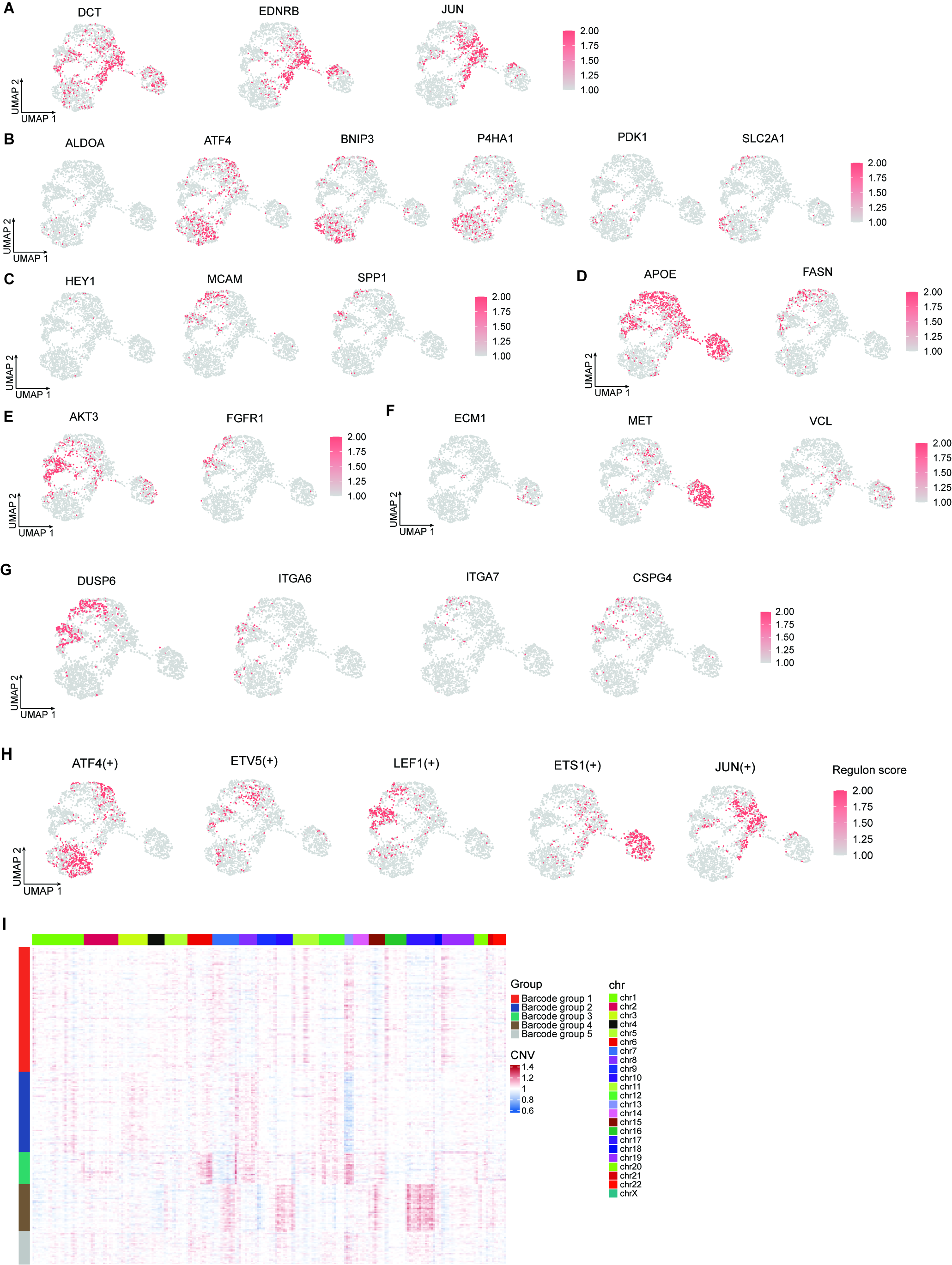
**

**Fig. S6 Genes and regulatory features associated with persister states in recurrent tumors.**

**(A)** UMAP showing expression of melanocytic markers DCT and EDNRB, together with the transcription factor JUN.

**(B)** UMAP showing expression of stress-like genes ALDOA, ATF4, BNIP3, P4HA1, PDK1, and SLC2A1.
**(C)** UMAP showing expression of NC-like genes HEY1, MCAM, and SPP1.

**(D)** UMAP showing expression of lipid metabolism-associated genes APOE and FASN.

**(E)** UMAP showing expression of PI3K signaling-associated genes AKT3 and FGFR1.

**(F)** UMAP showing expression of extracellular matrix (ECM) remodeling-associated genes ECM1, MET, and VCL.

**(G)** UMAPs showing expression of DUSP6, ITGA6, ITGA7, and CSPG4 within WM4237-1 barcoded subpopulations.

**(H)** SCENIC analysis showing regulon activity of transcription factors ATF4, ETV5, LEF1, ETS1, and JUN across barcode groups.

**(I)** Heatmap showing inferred copy-number variation profiles, with genes ordered by genomic position and cells grouped by barcoded subpopulations.


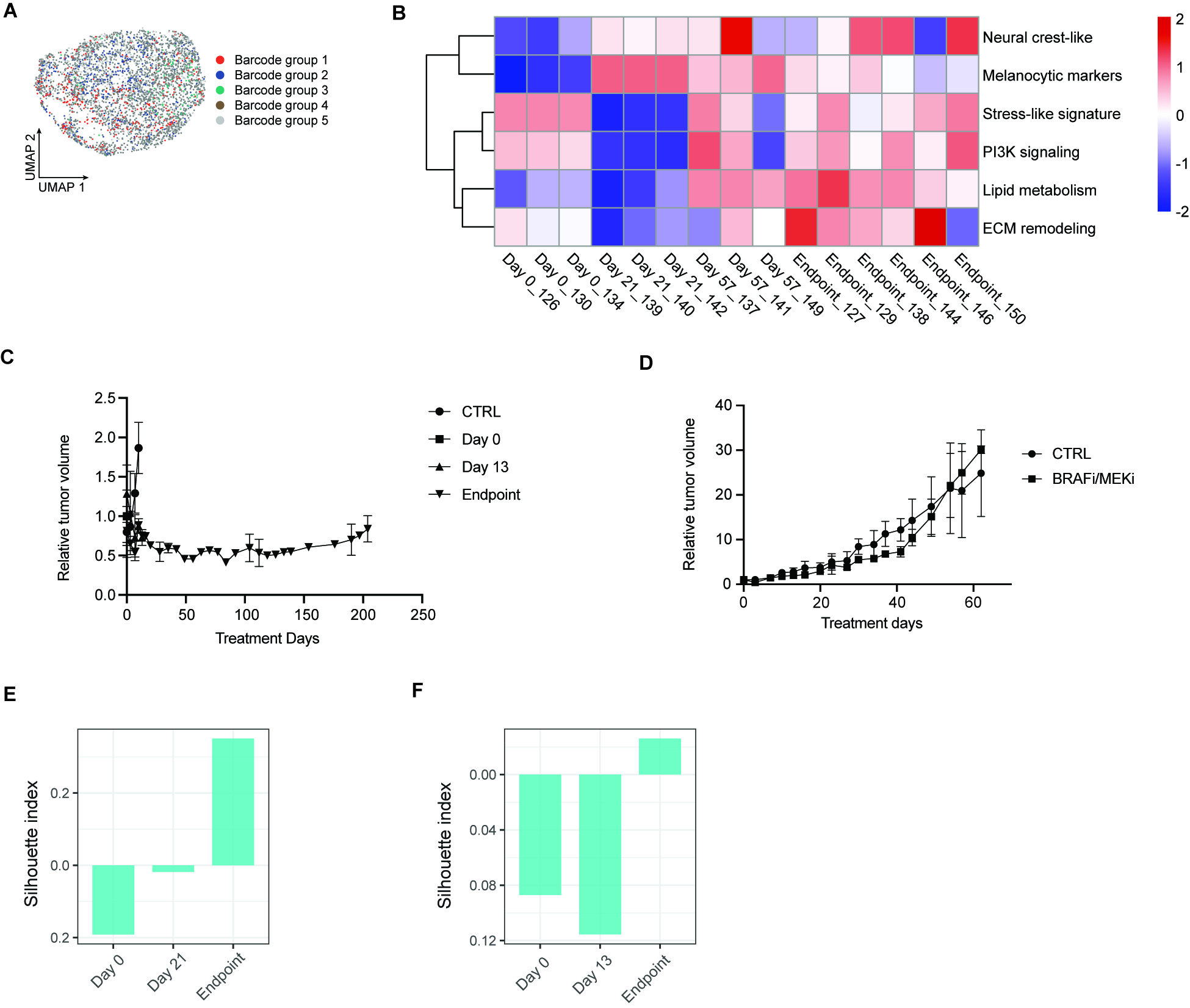


**Fig. S7 Persister programs are detected across treatment and PDX models.**

**(A)** ClonoCluster analysis applied to scRNA-seq data from pre-treatment (day 0) barcoded WM4237-1 tumors. UMAP showing projection of barcode groups 1-5, defined at the endpoint, onto the day 0 dataset.

**(B)** Heatmap showing activity of persister programs derived from bulk RNA-seq of barcoded WM4237-1 tumors across BRAFi/MEKi treatment.

**(C)** Tumor growth curves for the BRAFi/MEKi-sensitive PDX model WM4007, showing an initial response followed by relapse after approximately 7 months of treatment.

**(D)** Tumor growth curves for the intrinsically resistant PDX model WM4380-2, showing lack of measurable response to BRAFi/MEKi treatment.

**(E)** Silhouette index of persister programs across time points, quantified from ClonoCluster-derived UMAP embeddings of barcoded WM4237-1 scRNA-seq data.

**(F)** Silhouette index of persister programs across time points, quantified from UMAP embeddings of non-barcoded WM4007 scRNA-seq data.


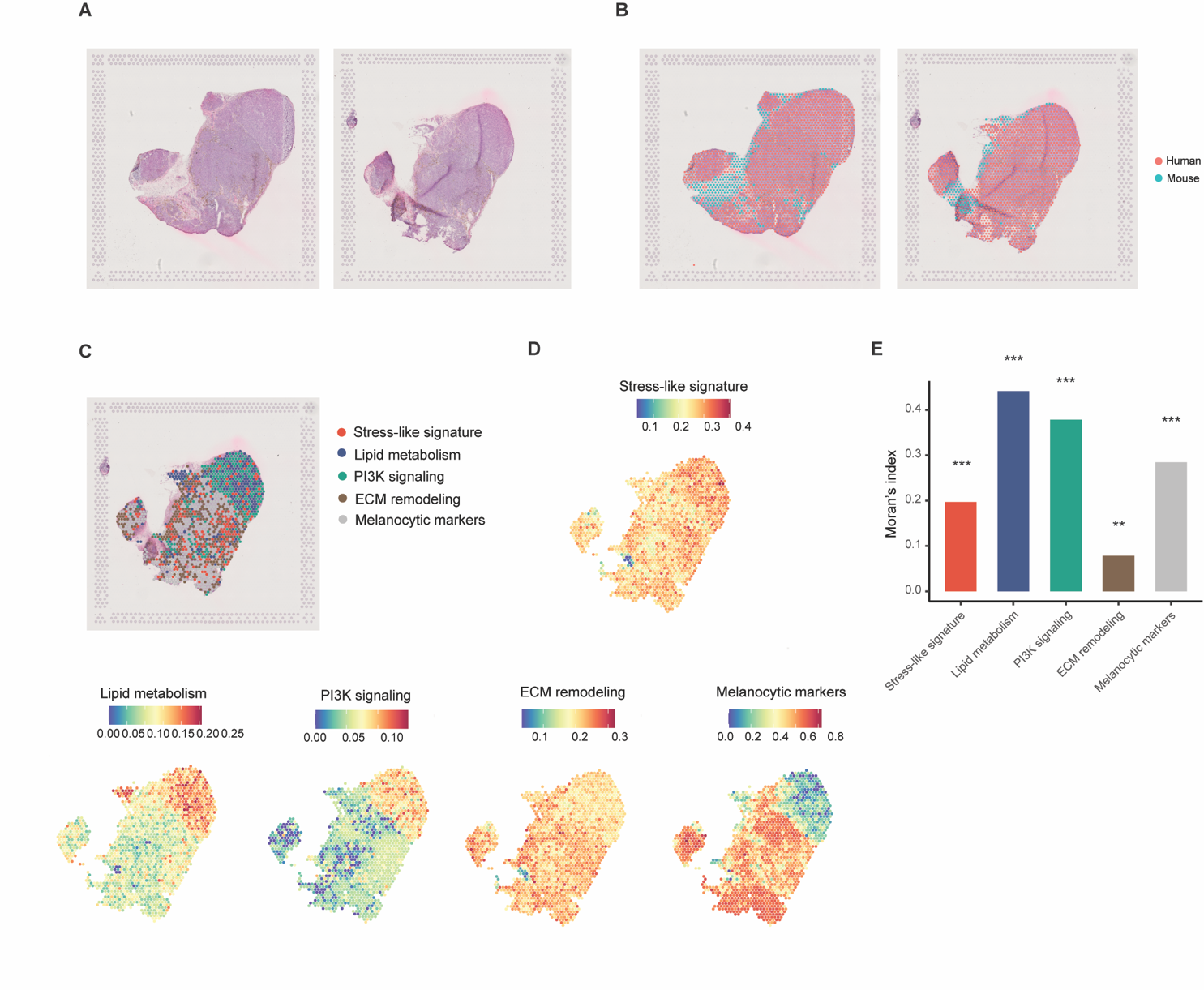


**Fig. S8 Spatial mapping of persister states in a recurrent WM4237-1 tumor.**

**(A)** Hematoxylin and eosin (H & E) staining showing histological morphology of two replicate sections from a WM4237-1 endpoint tumor.

**(B)** Spatial transcriptomics data showing the distribution of human tumor and mouse stromal reads on two replicate slides from a WM4237-1 endpoint tumor.

**(C)** Spatial transcriptomics visualization showing inferred transcriptional states overlaid on an H & E-stained section of a recurrent WM4237-1 PDX tumor, including stress-like (red), lipid metabolism (blue), PI3K signaling (green), ECM remodeling (brown), and melanocytic (grey) states.
**(D)** Spatial distributions of individual transcriptional states shown separately using the same color scheme as in panel (**C**).

**(E)** Moran’s index values showing spatial autocorrelation for each transcriptional state, including stress-like (red, I = 0.20), lipid metabolism (blue, I = 0.44), PI3K signaling (green, I = 0.38), ECM remodeling (brown, I = 0.078), and melanocytic (grey, I = 0.28). ** P < 0.01 and *** P < 0.001.


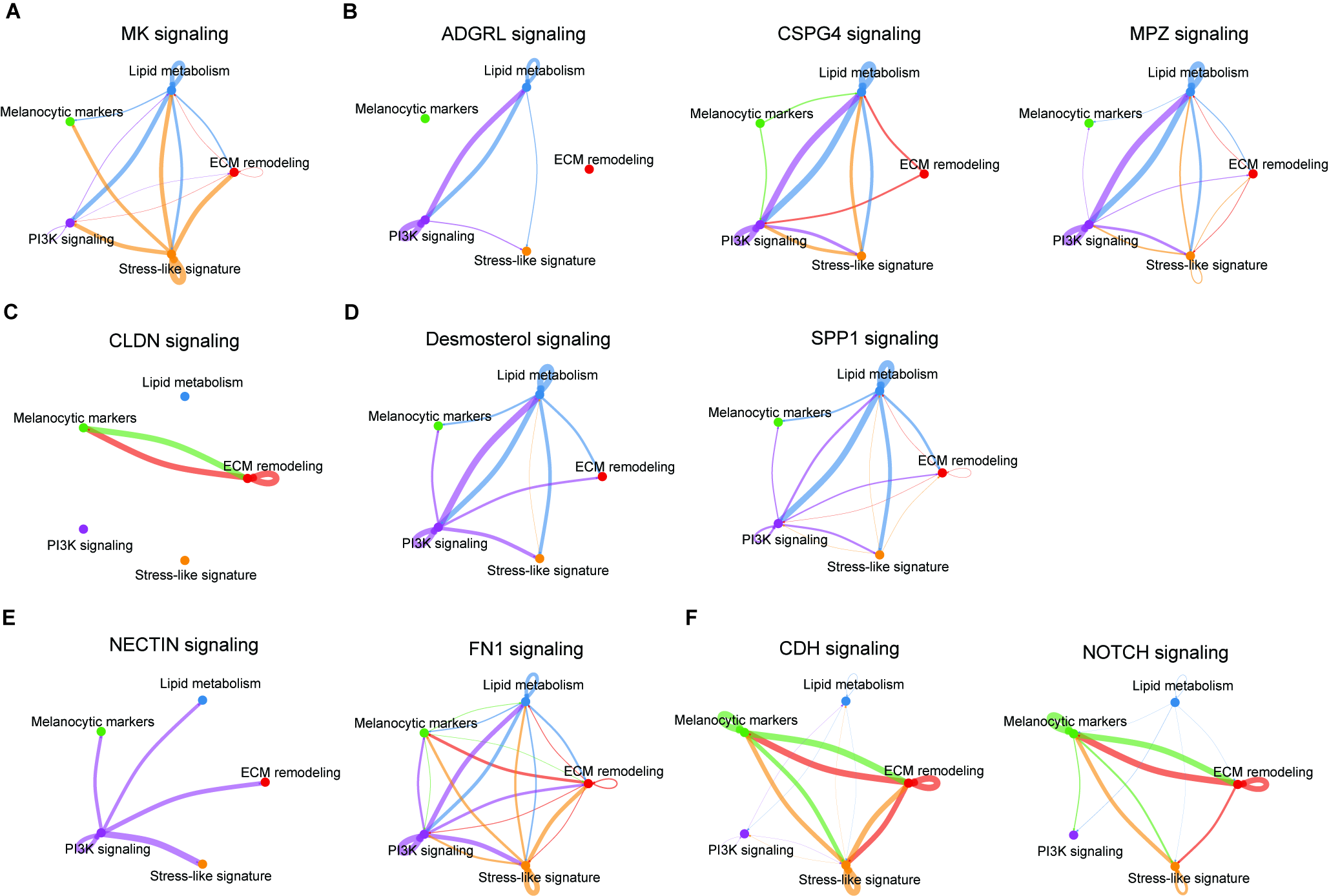


**Fig. S9 CellChat analysis of inferred cell-cell communication among persister states.**

**(A)** CellChat analysis showing inferred Midkine (MK) signaling originating from stress-like (yellow) and lipid metabolism (blue) states.

**(B)** Inferred signaling interactions involving ANGPTL*,* CSPG4, and MPZ between lipid metabolism (blue) and PI3K signaling (purple) states.

**(C)** Inferred Claudin (CLDN*)* signaling associated with the ECM remodeling state (red).

**(D)** Inferred Desmosterol and osteopontin (SPP1) signaling associated with the lipid metabolism state (blue).

**(E)** Inferred Nectin and fibronectin 1 (FN1) signaling associated with the PI3K signaling state (purple).

**(F)** Comparison of inferred Notch signaling (right) and cadherin (CDH) signaling (left), showing similar patterns of predicted cell-cell communication.
